# DNA methylation-calling tools for Oxford Nanopore sequencing: a survey and human epigenome-wide evaluation

**DOI:** 10.1186/s13059-021-02510-z

**Published:** 2021-10-18

**Authors:** Yang Liu, Wojciech Rosikiewicz, Ziwei Pan, Nathaniel Jillette, Ping Wang, Aziz Taghbalout, Jonathan Foox, Christopher Mason, Martin Carroll, Albert Cheng, Sheng Li

**Affiliations:** 1 The Jackson Laboratory for Genomic Medicine, Farmington, CT USA; 2 Present address: Center for Applied Bioinformatics, St. Jude Children’s Research Hospital, Memphis, TN USA; 3 Department of Genetics and Genome Sciences, UConn Health Center, Farmington, CT USA; 4 Department of Physiology and Biophysics, Weill Cornell Medicine, New York, NY USA; 5 The HRH Prince Alwaleed Bin Talal Bin Abdulaziz Alsaud Institute for Computational Biomedicine, Weill Cornell Medicine, New York, NY USA; 6 The Feil Family Brain and Mind Research Institute, New York, NY USA; 7 The WorldQuant Initiative for Quantitative Prediction, Weill Cornell Medicine, New York, NY USA; 8 Department of Medicine, University of Pennsylvania, Philadelphia, PA USA; 9 The Jackson Laboratory Cancer Center, Bar Harbor, ME USA; 10 Department of Computer Science and Engineering, University of Connecticut, Storrs, CT USA

**Keywords:** DNA methylation, Base modification, Long-read sequencing, Nanopore sequencing, Methylation calling

## Abstract

**Background:**

Nanopore long-read sequencing technology greatly expands the capacity of long-range, single-molecule DNA-modification detection. A growing number of analytical tools have been developed to detect DNA methylation from nanopore sequencing reads. Here, we assess the performance of different methylation-calling tools to provide a systematic evaluation to guide researchers performing human epigenome-wide studies.

**Results:**

We compare seven analytic tools for detecting DNA methylation from nanopore long-read sequencing data generated from human natural DNA at a whole-genome scale. We evaluate the per-read and per-site performance of CpG methylation prediction across different genomic contexts, CpG site coverage, and computational resources consumed by each tool. The seven tools exhibit different performances across the evaluation criteria. We show that the methylation prediction at regions with discordant DNA methylation patterns, intergenic regions, low CG density regions, and repetitive regions show room for improvement across all tools. Furthermore, we demonstrate that 5hmC levels at least partly contribute to the discrepancy between bisulfite and nanopore sequencing. Lastly, we provide an online DNA methylation database (https://nanome.jax.org) to display the DNA methylation levels detected by nanopore sequencing and bisulfite sequencing data across different genomic contexts.

**Conclusions:**

Our study is the first systematic benchmark of computational methods for detection of mammalian whole-genome DNA modifications in nanopore sequencing. We provide a broad foundation for cross-platform standardization and an evaluation of analytical tools designed for genome-scale modified base detection using nanopore sequencing.

**Supplementary Information:**

The online version contains supplementary material available at 10.1186/s13059-021-02510-z.

## Background

DNA methylation, the process by which methyl groups are added to DNA molecules, is a fundamental epigenetic modification process in gene transcription regulation [1]. Several DNA modifications, such as N6-methyladenine (6 mA), N4-methylcytosine (4mC), and 5-methylcytosine (5mC) and its oxidative derivatives, i.e., 5-hydroxymethylcytosine (5hmC), 5-formylcytosine (5fC), and 5-carboxylcytosine (5caC), are diversely distributed in genomes and play important roles in genomic imprinting, chromatin-structure modulation, transposon inactivation, stem cell pluripotency and differentiation, inflammation, and transcription-repression regulation [2–4]. DNA methylation measurement has traditionally depended on the combination of bisulfite conversion (which can damage DNA) and next-generation sequencing (which detects only short-range methylation patterns) [5].

Recently, third-generation sequencing technologies, including single-molecule real-time (SMRT) sequencing by Pacific Biosciences (PacBio), and nanopore sequencing by Oxford Nanopore Technologies (ONT), have overcome the read-length limitation to achieve ultra-long read, single-base detection at a genome-wide level [6, 7]. SMRT sequencing can detect 5mC modifications based on polymerase kinetics at 250× coverage [8]. However, this detection is not the result of direct 5mC detection at single-molecule resolution but rather the aggregation of the subtle impact of 5mC on polymerase kinetics signals during DNA synthesis [8]. Thus, the requirement for high coverage and inability of direct single-molecule 5mC detection by SMRT is a limitation [9]. In addition, while SMRT-based bisulfite sequencing allows sequencing of up to ~ 2 kilobases (kb) in length, it relies on bisulfite conversion [10].

**Fig. 1:**
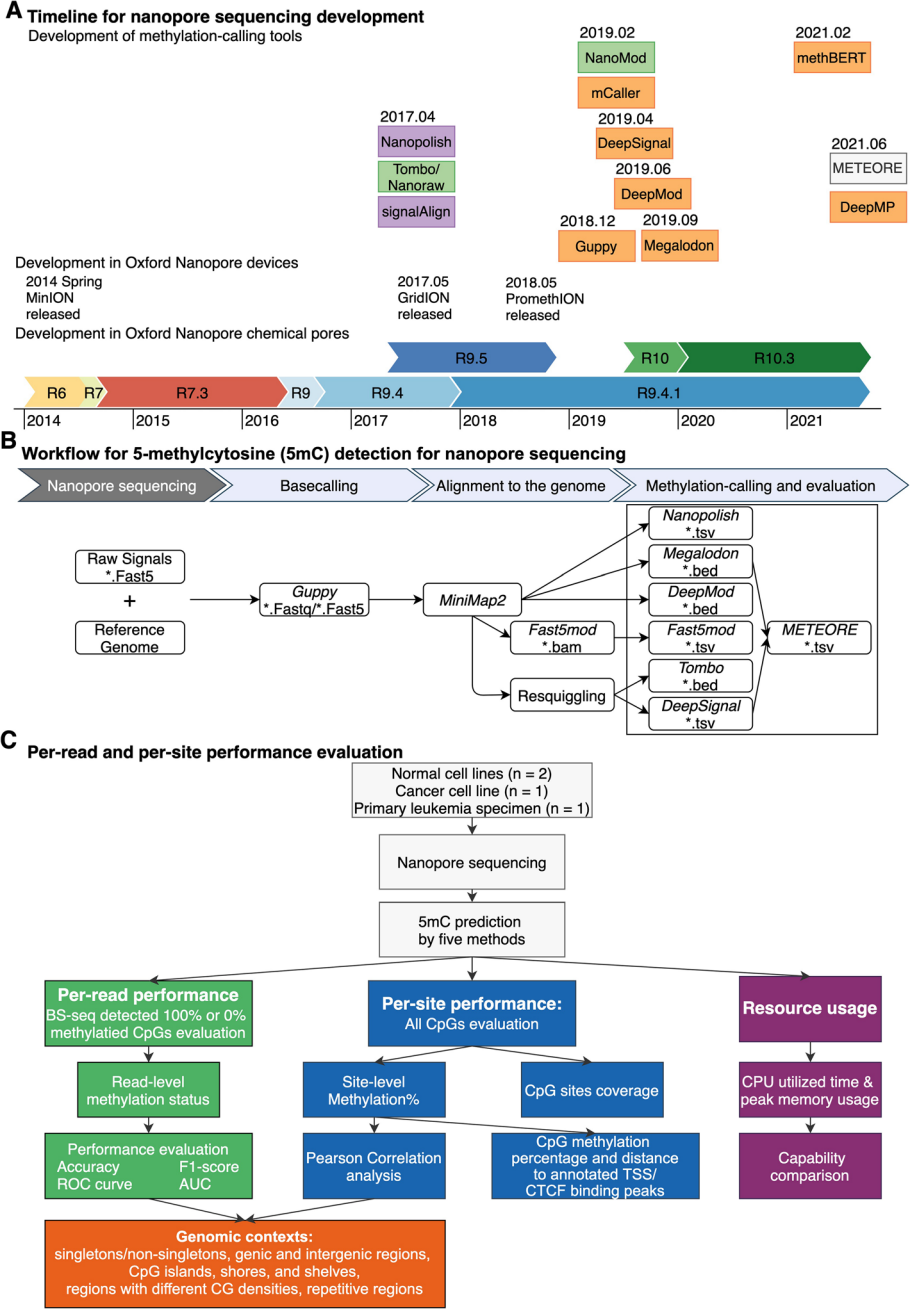
Technological development of methylation-calling tools and benchmark strategy. **A** Timeline of publication and technological developments of Oxford Nanopore Technologies (ONT) methylation-calling tools to detect DNA cytosine modifications. Methylation-calling tools are listed in the order of their publication dates instead of by their bioRxiv online submission dates (except for: BioRxiv date for methBERT and DeepMP, Github repository release time for Megalodon, since these two tools lack an available official publication). Chemical pore versions of Oxford Nanopore flow cells are represented as horizontal-colored bars. Methylation-calling tool are colored by the methylation calling methods (Green: statistical tests, Purple: HMM, Orange: neural network, white: machine learning models). Relevant publication dates are from multiple source [9, 12–23]. **B** Workflow for 5-methylcytosine (5mC) detection for nanopore sequencing. The analytic pipeline has three steps: (1) Basecalling by Guppy, which requires raw signals and reference genome as input. (2) Alignment to the reference genome by miniMap2 and re-squiggle by Tombo. (3) Methylation calling and evaluation. **C** Per-read and per-site performance evaluation. We considered the following genomic contexts: singletons, non-singletons, genic and intergenic regions, CpG islands, shores, and shelves, and regions with different CG densities, and repetitive regions. We utilized four nanopore sequencing benchmark datasets and BS-seq datasets as ground truth. We evaluated per-read, per-site performance, the running speed, and computing-memory usage

Nanopore sequencing, instead of using a sequencing-by-synthesis method to detect signal for the amplified DNA fragment population, is able to directly detect DNA or RNA translocation through a voltage-biased nanopore sensor, enabling rapid long-read sequencing and single-base, single-molecule sensitivity [11]. Several different versions of nanopore chemistry have been developed by ONT to improve the accuracy of single-molecule sequencing (Fig. 1A [9, 12–23]). Both the first pore version, termed R6 (“R” for Reader), and the subsequent R7 pore series yielded high error rates and only mediocre accuracy [11]. The next release, the R9 pore series, is derived from the bacterial amyloid secretion pore gene Curlin sigma S-dependent growth (CsgG) and yields a modal (i.e., most commonly observed) accuracy of up to 95% at the single-molecule level at higher sequencing speed [24, 25]. The accuracy of nucleobase identification in DNA sequencing can be measured using Q scores. These scores, also known as Phred quality scores, are logarithmically linked to the error probability (*P*) of each called base: *Q* =  − 10 × *log*_10_(*P*). Higher *Q* values correspond to lower error probability and higher quality [19, 26]. For example, Q30 indicates that the chance that a specific base is called incorrectly is 1 in 1000, and Q50 indicates that the chance is 1 in 100,000. The R9-series pores (including the original R9.4 version and the successor R9.4.1) are the most broadly used pore version, and R9.4.1 version can achieve > 99.99% (Q45) consensus accuracy [15, 27]. Recently, ONT released the R10 pore series, which has a predicted model accuracy of 94% [18, 28], and introduced the newest version, R10.3, which has a longer barrel and a dual-reader head inside the pore, with accuracies up to 95% and single-molecule consensus accuracies over Q50 [19, 29]. Our present study is conducted on the R9.4 and R9.4.1 version.

Nanopore sequencing techniques detect DNA modifications via differences in the electric current intensity produced from a nanopore read of an unmodified base and that of a modified base. Specifically, the electric current patterns, also known as “squiggles,” resulting from the passage of modified bases through the pores differs from the patterns produced by the passage of unmodified bases [26, 30]. The difference can be determined after nanopore read basecalling and alignment by (1) statistical tests comparing the electric current pattern to an in silico reference or the pattern from a non-modified control sample [20, 31]; (2) pre-trained supervised learning models, e.g., neural network [23, 32–37], machine learning model [38], and Hidden Markov Models (HMM) [9, 39]. However, DNA-methylation detection using nanopore sequencing presents a methodological challenge, i.e., the capacity to detect modifications in different CpGs that are in close proximity to one another on a DNA fragment (i.e., non-singleton), as it is assumed that all CpGs within a 10-bp region share the same methylation status. Twelve methylation-calling tools have been developed for various DNA modifications (e.g., 4mC, 5mC, 5hmC, and 6 mA) and for different nanopore pore versions (e.g., R7, R9, and R10) (Table 1 [9, 20, 23, 31–39]), but DNA-methylation detection for non-singletons containing both methylated and unmethylated CpGs remains difficult [9, 35]. Moreover, DNA methylation levels are not linearly distributed across the genome, and CpG density is dependent on genomic context [40–42]. Therefore, the accuracy of methylation callers likely differs among the different types of genomic regions within which the CpGs are located. Recent benchmarking work on methylation calling tools for nanopore sequencing either compared only three such tools and considered very few genomic contexts [43], or restricted the comparisons to *E. coli* and 1743 CpGs of the human genome [38]. Hence, there is no published guideline and systematic comparison of all current DNA methylation-calling tools for nanopore sequencing using natural human DNA [44], especially at the whole-epigenome scale. Recently, research with the combination of bisulfite-free enzymatic base conversion and nanopore sequencing [45–47] enabled high accuracy and potency in long-range epigenetic phasing. Together, these studies opened up new and orthogonal approaches to uncover the long-range coordination of epigenetic marks at single-molecule, single-base resolution.

**Table 1:** Current DNA methylation calling tools for Nanopore sequencing

Tools	DNA modification				Input required	Support multi-read FAST5 file?	Flow cells compatibility	Model trained on	Algorithm	Reported performance
	4mC	5mC	5hmC	6 mA						
Nanopolish [9]		✓			Basecalled FAST5	✓	R7.3, R9, R9.4, R9.4.1, R9.5, R10	*E. coli*	Hidden Markov model (HMM)	Accuracy = 0.94 (5mC, *Homo sapiens*)
Tombo/ Nanoraw [20]	✓	✓		✓	Raw FAST5		R9.4, R9.4.1, R9.5	no model	Mann-Whitney and Fisher’s exact test	Accuracy = 0.839, AUC = 0.78
SignalAlign [39]		✓	✓	✓	Basecalled FAST5		R7.3, R9.4^**a**^	Synthetic nucleotides	Hidden Markov model with a hierarchical Dirichlet process (HMM-HDP)	Accuracy = 0.76 (for 5hmC, 5mC)
								*E. coli*		Accuracy = 0.96 (for 5mC), Precision = 0.92
Guppy [32]		✓		✓	Raw FAST5	✓	R7.3, R9, R9.4, R9.4.1, R9.5, R10, R10.3	*Homo sapiens* and *E. coli*	Recurrent neural network	N/A
NanoMod [31]		✓			Basecalled FAST5, requires control sequence		R7.3, R9	no model	Kolmogorov-Smirnov test	Precision = 0.9
mCaller [33]				✓	Basecalled FAST5		R9, R9.4, R9.5	*E. coli*	Neural network	Accuracy = 0.954, AUC = 0.99
DeepSignal [35]		✓		✓	Basecalled FAST5 processed by Tombo re-squiggle module		R9, R9.4, R9.4.1	*E. coli*	Bidirectional RNN with LSTM+Inception structure	Accuracy = 0.92 (5mC, *Homo sapiens*), 0.90(m6A), Precision = 0.97
DeepMod [34]		✓		✓	FAST5 with raw signals and base calls		R9, R9.4, R9.4.1	*E. coli*	Bidirectional recurrent neural network (RNN) with long short-term memory (LSTM)	Precision = 0.99, AUC > 0.97
Megalodon [36]		✓		✓	Raw FAST5^**b**^	✓	R9.4.1, R10.3	*Homo sapiens* and *E. coli*^**c**^	Recurrent neural network^**d**^	N/A^**e**^
methBERT [23]		✓		✓	Raw FAST5		R9	*Homo sapiens* and *E. coli*	Bidirectional encoder representations from transformers (BERT)	Precision = 0.9147 (5mC, *Homo sapiens*)^**f**^
METEORE [38]		✓		✓	Methylation calling per-read results^**g**^		R9.4.1	*E. coli*	Random forest (RF), multiple linear regression (REG)	Root mean square error (RMSE) = 0.0687 (5mC, *E.coli*)^**h**^
DeepMP [37]		✓		✓	Basecalled FAST5 processed by Tombo re-squiggle module		R9, R9.4	*Homo sapiens, E. coli,* pUC19	Convolutional neural network (RNN)	F-score = 0.9324 (5mC, *Homo sapiens*)^**i**^

^a^SignalAlign’s compability on R9.4 is only validated on 5mC and 6 mA, not 5hmC

^b^Megalodon must obtain the intermediate output from the basecall neural network, and Guppy is the recommended backend to obtain this output from FAST5

^c^The model is trained in biological contexts only on *Homo sapiens* and *E. coli*. Users have to specify the model from the modified base models included in basecaller Guppy or research models in ONT Rerio repository

^d^Megalodon’s functionality centers on the anchoring of the high-information neural network basecalling output to a reference sequence

^e^The performance for Megalodon is not available since it is still under active development, no available published paper yet

^f^Only 5mC precision on *Homo sapiens* at per-site level is listed here, more performance parameter (AUC, Recall) of 5mC at per-site level and per-read level, and 5mC/6 mA performance on *E.coli* are available in the original paper

^g^METEORE combine the outputs from two or more methylation calling tools

^h^RMSE is for METEORE RF model combining Megalodon and DeepSignal at per-site level on selected sites of *E.coli*

^i^Only 5mC overall accuracy on *Homo sapiens* at per-read level is listed here. More performance parameters on *Homo sapiens*, and 5mC performance on *E.coli*, 5 mA performance on pUC19 plasmid are available in the original paper

Here, we present the first systematic benchmark of computational methods for detection of DNA 5mCs for nanopore sequencing at the human whole-genome scale. We assess the impact of CpG locations on detection accuracy using nanopore sequencing data generated from human cell lines and primary leukemia specimens, with a focus on the impact of singletons (CpG sites with only one CpG up and down 10-base-pair regions, Additional file 1: Fig. S1A), non-singletons (CpG sites with multiple CpG sites up and down 10-base-pair regions, Additional file 1: Fig. S1A), genomic context (i.e., genic and intergenic regions, CpG islands, shores, and shelves, Additional file 1: Fig. S1B), regions of various CG density, and repeat regions. Furthermore, even homogeneous cell populations can exhibit cell-to-cell variations in epigenetic patterns (epialleles), such as a gain or loss of cytosine methylation at specific loci [48]. Such epigenetic heterogeneity is increasingly recognized as a contributor to biological variability in tumors and worse clinical outcomes in malignancies [5]. Thus, to enable assessment of this critical epigenetic heterogeneity, we have evaluated the DNA methylation calling accuracy at single-molecule and single-base resolution, which is critical for epigenetic heterogeneity assessment [5, 48–50]. This comprehensive survey and systematic comparison offer user-specific, best-practice recommendations to maximize accurate 5mC detection using current methylation-calling tools and provides guidance for next-generation calling tools. We also generated and made available an R Shiny database to distribute the modification-detection power associated with different genomic regions using different tools, to assist in the development of future algorithms and analytic tools.

## Results

### Benchmark strategy

Currently, twelve analytic tools have been developed to detect DNA methylation using ONT direct sequencing (Table 1). Among them, ten tools are compatible with R9.4 series flow cells, and nine of these ten can predict 5-methylcytosine (5mC). We compared the performance of those seven state-of-the-art methylation-calling tools targeting 5mCs in different CpG contexts; those seven tools are all compatible with the most favored ONT flow cell version (R9.4 and R9.4.1 pores): Nanopolish [9], Megalodon [36], DeepSignal [35], Guppy [32, 51], Tombo/Nanoraw (referred to as Tombo) [20], DeepMod [34], and METEORE [38] (Fig. 1B). Tombo is statistics-based while the other six tools are model-based. METEORE combines predictions from two or more tools that showed improved accuracy over individual tools using random forest (RF) models or multiple linear regression models. We chose the METEORE RF model combining Megalodon and DeepSignal as it achieved lower root mean square error (RMSE) than other available METEORE models [38]. We excluded SignalAlign [39], as its repository has not been updated for over 4 years. We also excluded DeepMP [37], as its repository is still under development. We developed the following three-step standardized workflow for benchmarking (Fig. 1B, C):

#### Step 1. Basecalling and quality control

**Fig. 2:**
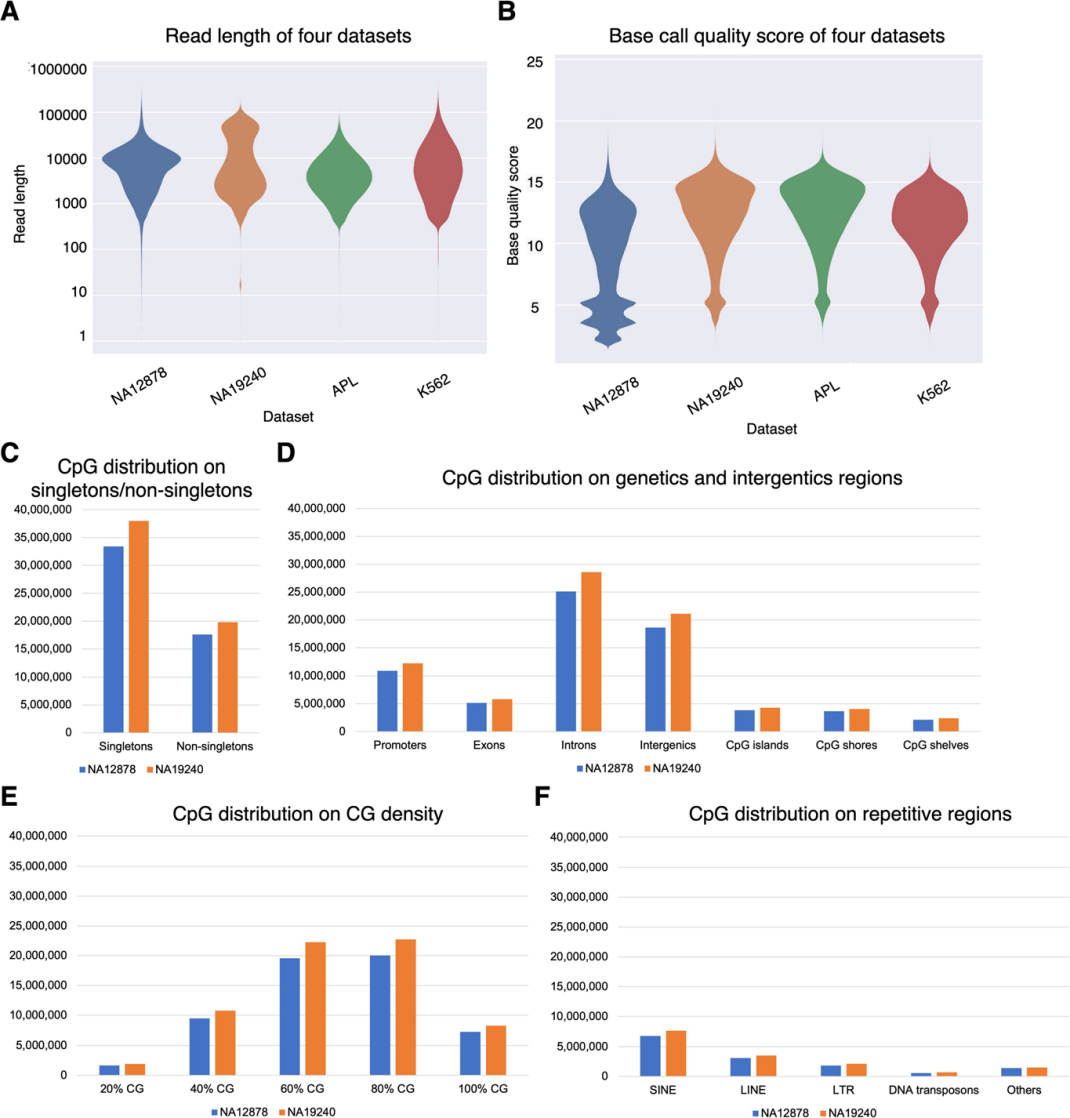
Characteristics of the nanopore sequencing datasets. **A, B** Quality assessment for four datasets. **A** Violin plot of read length. **B** Violin plot of basecalling quality. Data shown are colored by dataset and plotted by Plots NanoPack [52]. **C–F** CpG distribution (coverage ≥ 3) of human B-lymphocyte cell line NA19240 and NA12878 nanopore sequencing data based on **C** singletons/non-singletons, **D** genic and intergenic regions, **E** different CG densities, and **F** repetitive regions

To translate raw signal data into nucleotide sequences, we conducted the basecalling step using Guppy (v4.2.2). Then we used NanoPack [52] for data visualization and processing, to assess the read-length and basecalling quality, and to demultiplex sequencing data for downstream analysis. Together, the four ONT datasets exhibited median read lengths ranging from 3756 to 6524 bp, and median base quality ranging from 9.8 to 13 (Fig. 2A, B). The proportion of long reads (> 10,000 bp) is higher in the NA19240 dataset (36.75%) than in the other three datasets (median proportion = 32.29%), due to library preparation differences (See “Methods” for more details). We assessed CpG sites located in singletons and non-singletons (Additional file 1: Fig. S1A), and biologically relevant genomic contexts including gene bodies and CpG islands (Additional file 1: Fig. S1B), different CpG densities, and repetitive regions. The distribution of CpG sites in different regions is shown in Fig. 2C–F, Additional file 1: Fig. S2, and Additional file 2: Table S1.

#### Step 2. Genome assembly and polishing

We aligned the basecalled reads to human genome assembly GRCh38/hg38 using minimap2 [53]. Basecalling a squiggle, i.e., translating the electric current signal from a nanopore read into a DNA sequence, typically contains some errors when comparing the resulting sequence to a reference sequence [54]. The Tombo re-squiggle algorithm refines the assignment from a squiggle to a reference sequence after basecalling and alignment. The refined basecalled reads and alignment by this re-squiggle algorithm is required by Tombo and DeepSignal for DNA methylation calling.

#### Step 3. Methylation calling and evaluation

We detected 5mCs in different CpG contexts using each of the seven methylation-calling tools based on the corresponding recommended parameters. Specifically, Guppy recommended using the ONT fast5Mod program [55] to extract the methylation calling information at the site level from the basecalling output (Fig. 1B). We then designed three performance-evaluation criteria (Fig. 1C) to benchmark the performances of each methylation-calling tool and used bisulfite sequencing (BS-seq, coverage ≥ 5) data to determine the ground truth. First, we evaluated the *per-read* performance of the 5mC prediction, i.e., at single-molecule, single-base resolution, based on fully methylated or fully unmethylated CpG sites across various genomic contexts. The performance metrics included F1 score, accuracy, receiver operating characteristic curves (ROC curves), and area under the ROC curve (AUC). Second, we assessed the *per-site* performance of the 5mC prediction. Specifically, we measured the 5mC percentage correlation coefficient between nanopore sequencing and BS-seq across all CpG sites at the human whole-genome level. Furthermore, we evaluated the relationship between CpG methylation percentage and distance to the annotated transcription start site (TSS) or CCCTC-binding factor (CTCF) binding sites. Third, we assessed the running speed and resource usage evaluation. Further details on performance criteria used in the evaluation are shown in “Methods.”

### Benchmark datasets

We used four datasets for benchmarking: nanopore sequencing of the human B-lymphocyte cell lines NA19240 (referred to as NA19240, R9.4.1) [56] and NA12878 (referred to as NA12878, R9.4) [57], the human leukemia cell lines K562 (referred to as K562, R9.4.1), and a human primary acute promyelocytic leukemia clinical specimen (referred to as APL, R9.4.1).

For nanopore sequencing, we used published high-coverage nanopore sequencing datasets for the cell line NA19240 (~ 32× sequencing coverage) from the 1000 Genomes Project [56], and the cell line NA12878 (~ 26× sequencing coverage) from Whole Human Genome Sequencing Project [57], and generated nanopore sequencing datasets for K562 and APL with ~ 1–3× coverage. For DNA methylation ground truth, we used the published NA12878 and K562 whole-genome bisulfite sequencing (WGBS) datasets, and the NA19240 reduced representation bisulfite sequencing (RRBS) dataset from the Encyclopedia of DNA Elements (ENCODE) [58]. We also generated WGBS and oxidative bisulfite sequencing (oxBS-seq) data for APL. More details can be found in “Methods.”

### Per-read performance of 5mC prediction

**Fig. 3:**
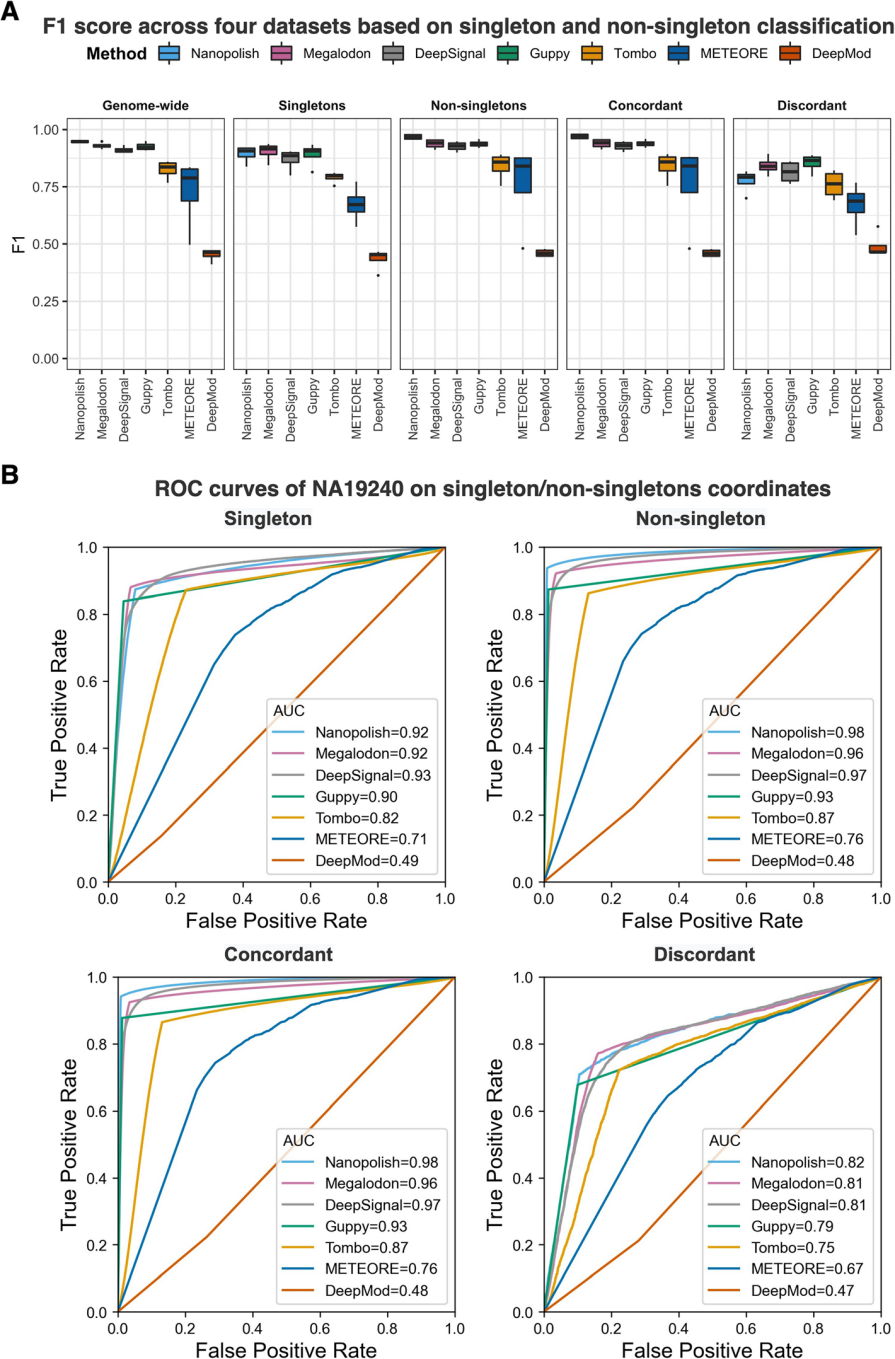
Per-read performance of 5mC prediction at singletons and non-singletons. **A** F1 score across four datasets based on singleton and non-singleton classification using BS-seq as ground truth. Singletons are CpG sites that contain only one CpG up and down 10-base-pair (bp) regions; non-singletons are CpG sites with multiple CpG sites up and down 10-bp regions; concordant non-singletons are non-singletons where all CpGs with a 10-bp region have the same methylation state (i.e., all 100% or all 0% methylated); discordant non-singletons are non-singletons with both fully methylated and fully unmethylated CpGs. **B** ROC curves for the NA19240 dataset on singletons, non-singletons, concordant non-singletons, and discordant non-singletons

Nanopore sequencing can detect cytosine-methylation state for individual molecules. We assessed the per-read performance of the seven DNA-methylation-calling tools at single-molecule, single-base resolution in singletons and non-singletons. We compared methylation calling performances on fully methylated or fully unmethylated CpGs using BS-seq as ground truth across the four datasets (Additional file 2: Table S2). We divided non-singletons into two sub-categories: (1) concordant non-singletons: non-singletons contain CpGs that are either fully methylated or fully unmethylated, and (2) discordant non-singletons: non-singletons that contains both fully methylated and fully unmethylated CpGs. Nanopolish, Megalodon, DeepSignal, and Guppy outperformed the other three tools on all datasets measured by F1-score, accuracy, and AUC (Fig. 3, Additional file 1: Fig. S3, and Additional file 2: Table S3). Notably, all tools exhibited lower F1 scores (less than 0.90, Fig. 3A) and accuracy (less than 0.93, Additional file 1: Fig. S3B) at discordant non-singletons than at any of the other CpG contexts, consistently across four datasets (Additional file 1: Fig. S3C). Also, all methods achieved higher performance on concordant non-singletons than singletons. The observation may be relevant to the fact that model-based methylation-calling tools (e.g., Nanopolish, DeepSignal, DeepMod, and METEORE) used “concordant” training data—completely methylated sequences and completely unmethylated sequences. Moreover, Nanopolish and Tombo borrow the signals of neighboring CpG sites to call DNA methylation.

**Fig. 4:**
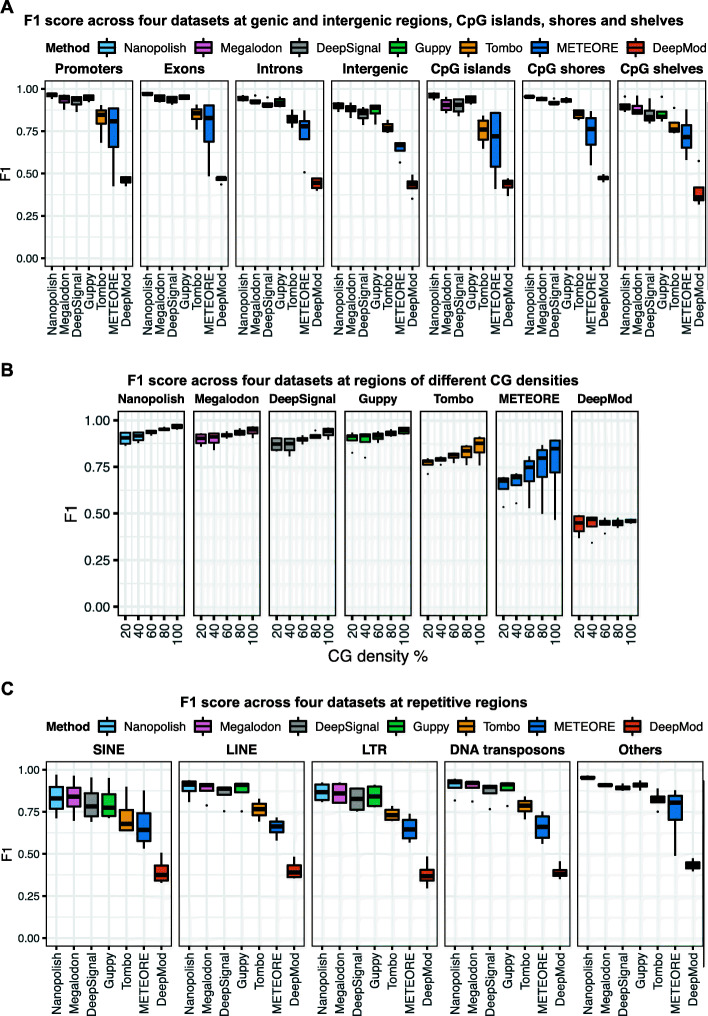
Per-read performance of 5mC prediction in different genomic contexts. **A** F1 score across four datasets at genic and intergenic regions, CpG islands, shores, and shelves. Promoter is 2000 bp up and down the transcription start site (TSS). **B** F1 score across four datasets at regions of different CG densities (20%, 40%, 60%, 80%, 100%). **C** F1 score across four datasets at repetitive regions. We consider short interspersed nuclear elements (SINE), long interspersed nuclear elements (LINE), long terminal repeats (LTR), DNA transposons, and “Others” for other repetitive regions. **A–C** Evaluation across four datasets using BS-seq as ground truth

Different genomic contexts display different CpG densities and DNA methylation levels [59]. Thus, to evaluate the impact of biologically relevant genomic contexts on 5mC predictions, we considered promoters, exons, introns, intergenic regions (referred as intergenic), CpG islands, shores, and shelves (Fig. 4A, Additional file 1: Fig. S4A, Additional file 2: Table S3), regions with different CG densities (Fig. 4B, Additional file 1: Fig. S4B), and different types of repetitive regions (Fig. 4C, Additional file 1: Fig. S4C). All seven tools exhibited a lower F1 score (< 0.93) for intergenic regions than for any other genic regions or CpG islands, shores, and shelves (Fig. 4A). We next assessed if CG density impact the performance of 5mC predictions using nanopore sequencing (Fig. 4B). Specifically, CG density is calculated by the percentage of G and C bases in 5-base windows. Tombo and METEORE suffered from low accuracy predictions in all CG density regions, but particularly so in low CG density regions. CG density significantly associated with the performance of Nanopolish, Megalodon, DeepSignal, Guppy, and Tombo with *p* value < 0.05 by the analysis of variance (ANOVA) test (Additional file 2: Table S4). Moreover, we examined five categories of repetitive regions: short interspersed nuclear element (SINE), long interspersed nuclear element (LINE), long terminal repeat (LTR), DNA transposons, and others (Fig. 4C). Nanopolish, Megalodon, DeepSignal, Guppy, and Tombo showed lower F1 scores for SINE and LTR regions than for the other repetitive regions. Compared to the other tools, Nanopolish, Megalodon, DeepSignal, and Guppy consistently exhibited higher overall F1 scores on CpG sites across all datasets and across genic and intergenic regions, repetitive regions, and regions with different CG densities (Fig. 4).

DeepMod ranked the lowest in F1 score, accuracy, and AUC, when applied to all four *human* ONT datasets (Additional file 2: Table S3) across different genomic contexts (Figs. 3 and 4), while it is comparable to the other six tools when using the 5mC positive control dataset from *E. coli* [38] (Additional file 2: Table S5), suggesting the importance of evaluating the performance of these analytic tools using human ONT datasets, since not all tools are compatible with genomes with higher complexity than that of *E. coli*, such as the human genome.

To determine whether the performance of the tools at genomic regions in general is impacted by the percentage of non-singletons in the regions, we assessed the percentage of singletons and non-singletons for each genomic context (Additional file 2: Table S6). In addition, we tested the significance of the Pearson correlation coefficient between F1 score achieved by each tool and non-singleton percentage, and the relationship was statistically significant (*p* value < 0.05, correlation coefficients range from 0.286 to 0.423, Additional file 2: Table S7) for three tools, i.e., Nanopolish, DeepSignal, and Guppy. These observations suggest that the various 5mC prediction performances across different genomic contexts are significantly influenced by the distribution of singletons and non-singletons in three tools. In summary, Nanopolish, Megalodon, DeepSignal, and Guppy outperformed the other tools in per-read performance of 5mC prediction across genomic contexts.

### Per-site performance of 5mC prediction

**Fig. 5:**
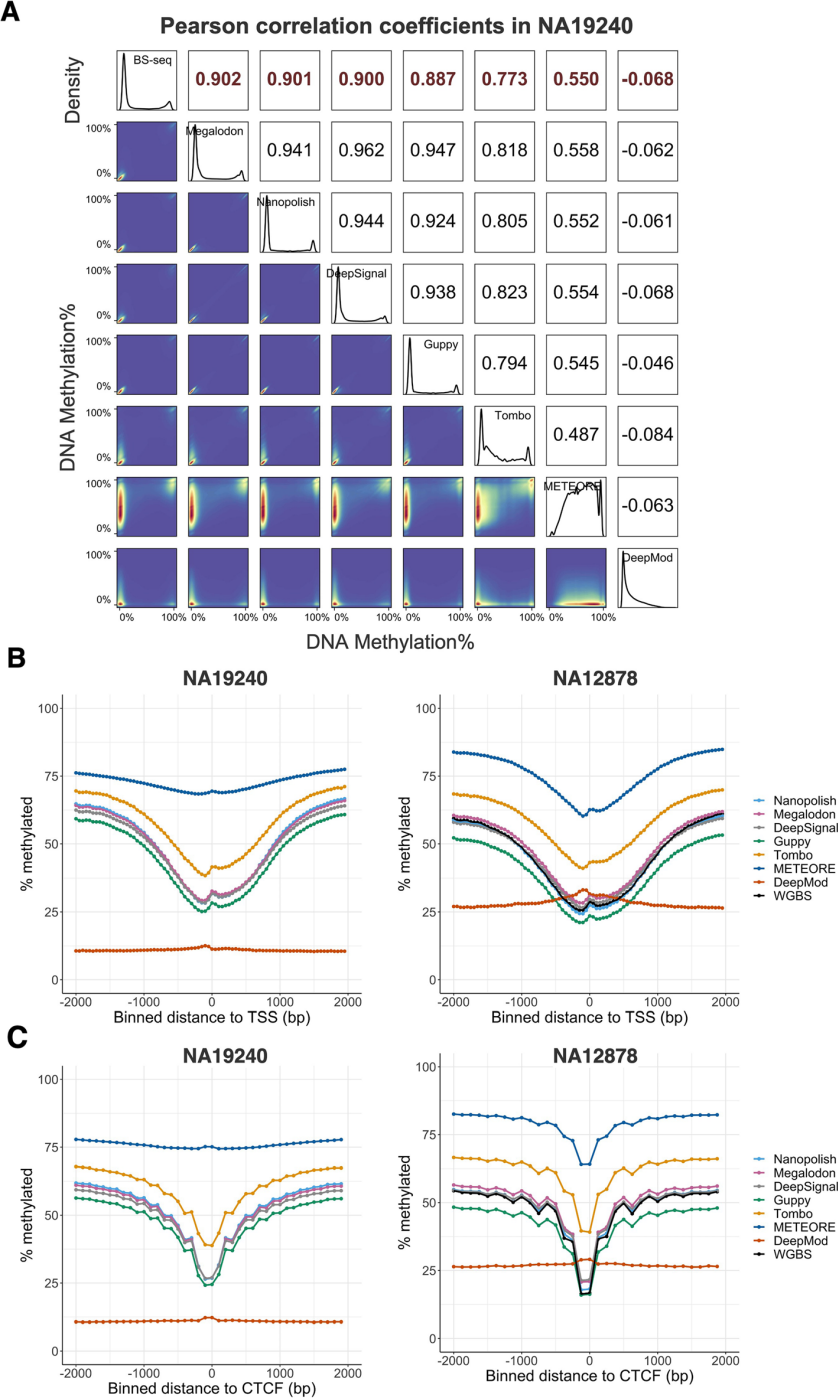
Per-site performance of 5mC prediction. **A** Correlation plot showing Pearson correlation coefficients of each methylation-calling tool with BS-Seq on NA19240. The white squares in the upper triangle show Pearson correlation coefficients. Cross-platform (i.e., between BS-seq and nanopore sequencing) correlation coefficients are in red while cross-tool (i.e., pair-wise comparison between each methylation-calling tool for nanopore sequencing) correlation coefficients are in black. The density plots in the diagonal exhibit the distributions of 5mC percentage. The blue squares in the lower triangle represent the 2D kernel density plots for each pair of comparisons. **B** Relationship between CpG methylation percentage and distance to annotated TSS in the NA19240 (left panel) and NA12878 (right panel) datasets. Distances are binned into 50-bp windows. **C** Relationship between CpG methylation percentage and distance to annotated CTCF binding peaks in the NA19240 (left panel) and NA12878 (right panel) datasets. Distances are binned into 100-bp windows for NA19240 and 125-bp windows for NA12878. Negative distances are upstream and positive distances are downstream of the **B** TSS or **C** CTCF binding peaks

To assess the performance of the seven tools for CpG sites with a full range of methylation levels, we evaluated the Pearson correlation coefficient between DNA methylation percentage from nanopore sequencing (read coverage ≥ 3) and from the corresponding BS-seq data (coverage ≥ 5), both at single-base resolution. To obtain per-site DNA methylation percentages, we either obtained DNA methylation reports directly from each tool or followed the instruction of each tool to aggregate per-read 5mC predictions or to obtain the fraction of methylated reads. We found that the 5mC percentage predicted by Nanopolish, Megalodon, DeepSignal, and Guppy showed the highest correlation (≥ 0.80) with all four datasets (Fig. 5A and Additional file 2: Table S8).

BS-seq of all datasets exhibited a bimodal distribution of DNA methylation (0 for unmethylated, 1 for methylated), and the histogram of the DNA methylation output of Nanopolish, Megalodon, DeepSignal, and Guppy also displayed a similar bimodal distribution on NA19240 (Fig. 5A). Similar predicted methylation patterns and performance of Nanopolish, Megalodon, and DeepSignal for NA12878 were observed by recent research [43]. In contrast, the DNA methylation-level histogram of Tombo and METEORE showed multiple peaks between 0 and 100% methylation levels, rather than two peaks. Furthermore, the Pearson correlation between BS-seq and DeepMod was close to zero for NA19240 data (Fig. 5A), confirming that DeepMod cannot effectively predict methylation distribution at the human whole-genome level. Moreover, Nanopolish, Megalodon, DeepSignal, and Guppy consistently produced the highest correlation coefficients at all genic and intergenic regions, CG density regions, and repetitive regions for NA19240 data (Additional file 1: Fig. S5A-B).

Furthermore, we found that the correlation among the top four performers, i.e., Guppy, Nanopolish, Megalodon, and DeepSignal, is greater than the correlation between BS-seq and nanopore sequencing data (Fig. 5A, Additional file 2: Table S8). We hypothesize that the ability to distinguish 5hmC from 5mC by nanopore sequencing, at least in part, contributes to the discrepancy between BS-seq and nanopore sequencing. To test the hypothesis, we generated oxBS-seq for APL (See “Methods”) and detected the 5hmC percentage for each CpG site by integrating matched APL oxBS-seq and WGBS data by the MLML (maximum likelihood methylation levels) algorithm [60]. We compared the 5hmC percentage in CpGs exhibiting agreement (methylation level difference < 5%) and discrepancy (methylation level difference > 40%) between WGBS and nanopore sequencing for APL. The CpG sites exhibiting discrepancy showed a significantly higher 5hmC level than those exhibiting agreement (*p* value < 0.0001, Wilcoxon rank sum test, Additional file 1: Fig. S6A-D). These observations suggest that the ability to distinguish 5hmC from 5mC by nanopore sequencing enables a more accurate 5mC detection than BS-seq.

To assess the impact of biological context on the methylation predictions, we explored the relationship between CpG methylation percentage and the distance to annotated TSSs. As expected, CpG sites near TSSs tended to be unmethylated. Methylation levels increased as the distance from the TSS increased. DNA methylation patterns detected from nanopore sequencing via Nanopolish, DeepSignal, and Megalodon closely resembled the pattern for the WGBS data (Fig. 5B, Additional file 1: Fig. S7A-B, and Additional file 2: Table S9). Notably, Guppy displayed the lowest DNA methylation levels at TSSs. Furthermore, methylated cytosines affect DNA-binding specificities of hundreds of human transcription factors [61]. Binding sites of the transcription factor CTCF are characterized by low DNA methylation levels [62]. CTCF plays a critical role in long-range chromatin interactions, the formation and maintenance of topologically associated domains, and transcription. Thus, we assessed the relationship between CpG methylation percentage and the distance to the center of the CTCF binding peaks from the ChIP-seq data of the matching cell lines (NA19240, NA12878, and K562). Indeed, DNA methylation percentage was lowest at the center of the CTCF binding peaks, and the ONT 5mC predictions by Nanopolish, Megalodon, DeepSignal, and Guppy closely tracked the pattern of WGBS data (Fig. 5C, Additional file 1: Fig. S7C, and Additional file 2: Table S9).

Overall, Nanopolish, Megalodon, DeepSignal, and Guppy had high correlations with BS-seq, and they closely tracked the methylation patterns of BS-seq at the whole-genome level. METEORE, which models 5mC by integrating the output of Megalodon and DeepSignal, did not perform well in any evaluation criteria that we assessed. The correlation coefficient of DNA methylation across CpG sites between the seven tools and BS-seq is consistent with the read-level accuracy (Figs. 3 and 4).

### Megalodon and DeepSignal predicted more CpG sites than did Nanopolish and Guppy at the site level

**Fig. 6:**
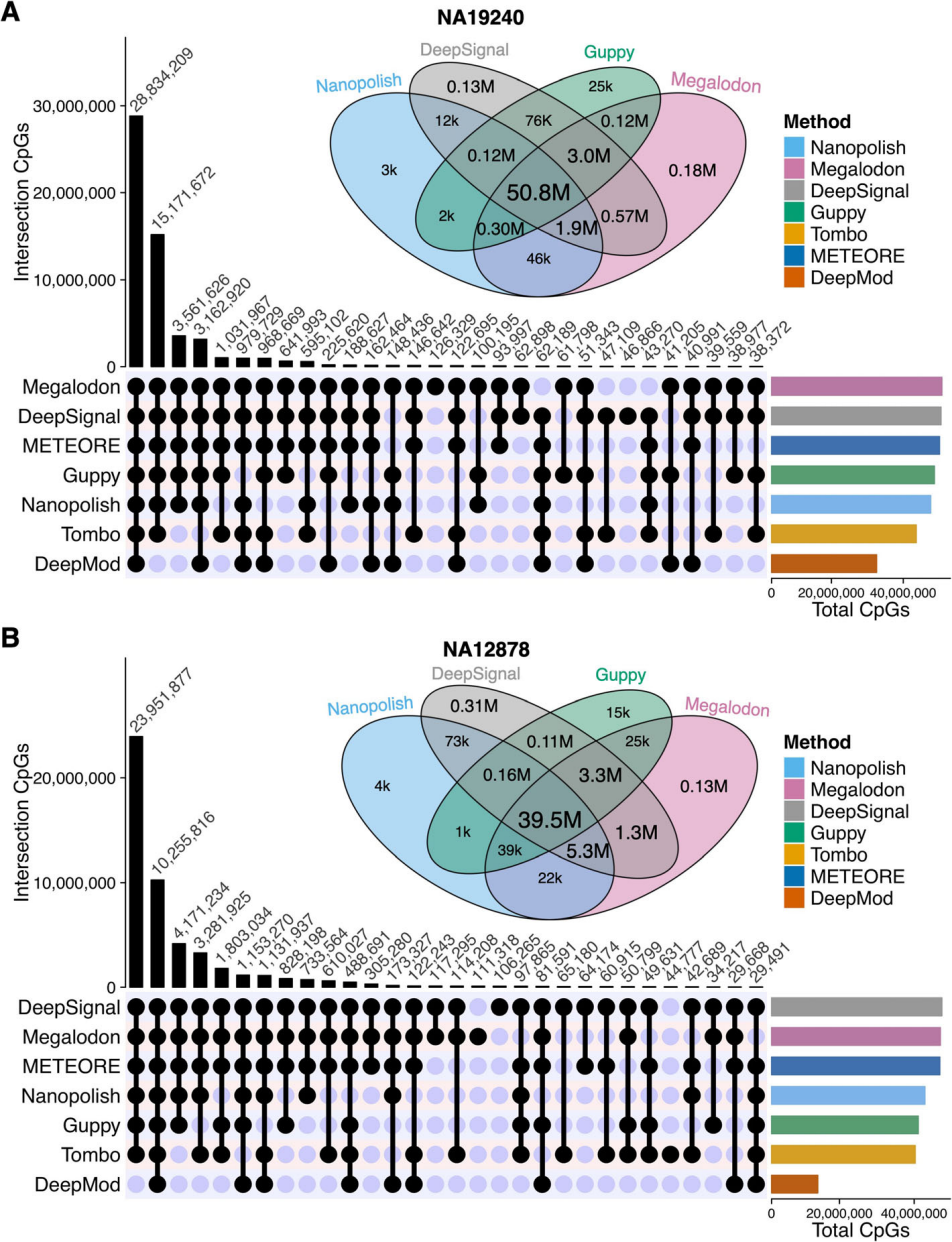
The overlap of CpG sites predicted by methylation-calling tools. UpSet diagram shown at bottom of each panel (**A** and **B**) is for the CpG sites of the top thirty sets of intersections detected by each methylation-calling tool using (**A**) NA19240 and (**B**) NA12878 nanopore sequencing datasets. Venn diagram shown at the top of each panel (**A** and **B**) is for CpG sites detected by the four best-performing methylation-calling tools (Nanopolish, DeepSignal, Guppy, Megalodon), k is for thousand and M is for million. The numbers of CpGs in each intersection of Venn diagram can be found in Additional file 2: Table S11. Bar plot shown at the lower right of each panel (**A** and **B**) is for the total CpGs detected by each tool. For each methylation-calling tool, only the CpG sites covered by ≥ 3 reads were considered

Though CpG sites are the same for all the tools after the basecalling and alignment step, the predicted number of CpG sites is different because each methylation-calling tool has their own criteria to make confident methylation predictions. Therefore, we next evaluated the number of CpG sites (read coverage ≥ 3) with 5mC predictions. The UpSet diagram shows the number of overlapped sites among the tools (Fig. 6 and Additional file 1: Fig. S8). Compared to the other five tools, Megalodon and DeepSignal covered more CpG sites on all four datasets. Of the predicted CpG sites in NA19240, 50% of the sites were predicted by all tools (Additional file 2: Table S10). Furthermore, among all the CpG sites predicted collectively by the top four performers (i.e., Nanopolish, Megalodon, DeepSignal, and Guppy), 92% and 85% of the sites were predicted by all four tools for the NA19240 and NA12878 datasets, shown by the Venn diagrams (Fig. 6 and Additional file 2: Table S11). Megalodon and DeepSignal predicted the highest number of CpG sites, i.e., they each predicted 99% of the union of CpG sites using the NA19240 dataset, while Nanopolish and Guppy predicted 93% and 95%, respectively, of the union CpG sites in that dataset, due to the more stringent criterion of log-likelihood ratio cutoffs [9, 32, 51, 63]. Thus, Megalodon and DeepSignal covered 6% and 4% more CpG sites than did Nanopolish and Guppy, and the differences increase greatly for lower sequencing-depth ONT datasets (APL and K562, Additional file 1: Fig. S8A-B). In summary, among the top four performers, Megalodon and DeepSignal predicted the largest number of CpG sites. Also, Tombo and DeepMod predicted the fewest CpG sites.

### Running time and memory usage on benchmark datasets

**Fig. 7:**
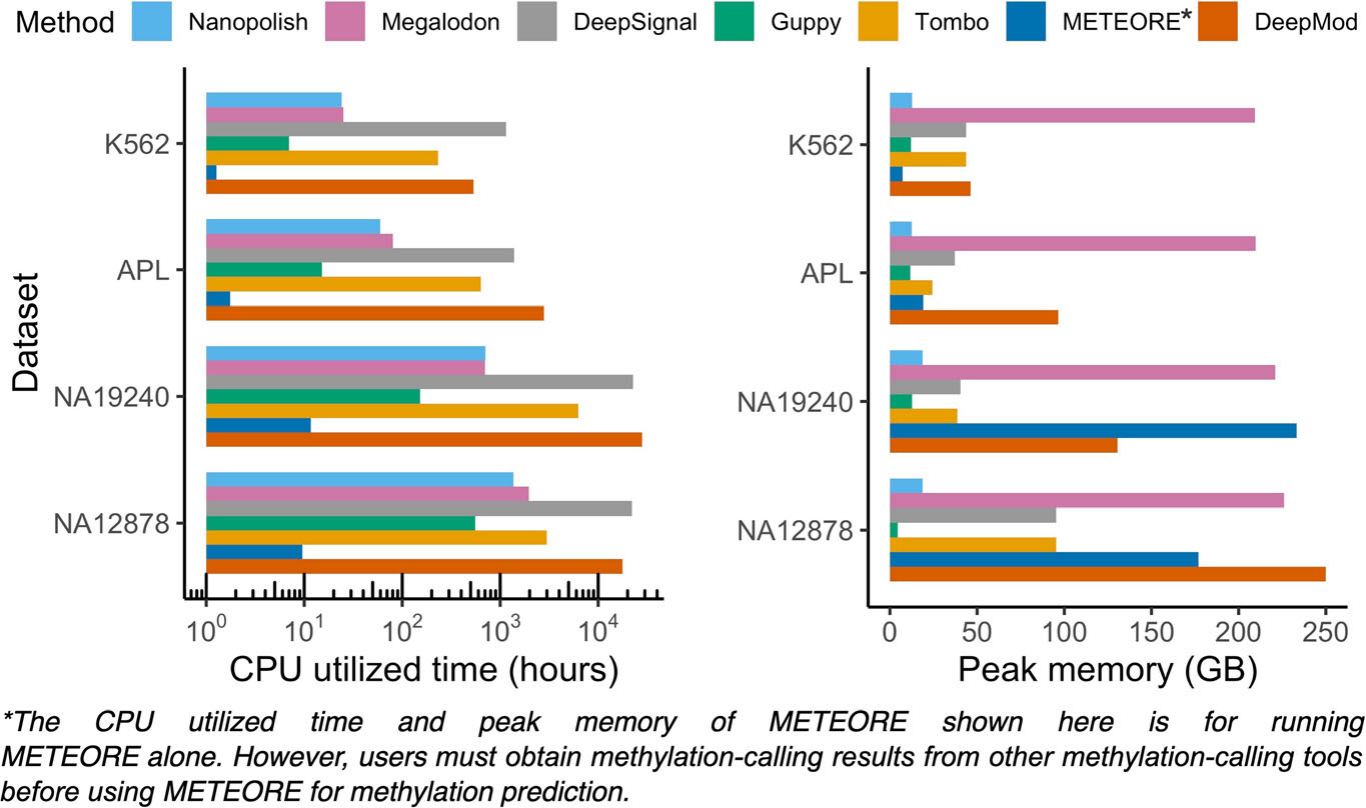
CPU utilized time and peak memory usage. We compared the peak memory usage and running time of the seven tools on the single-read fast5 files of each dataset

To evaluate the running time and peak memory of each methylation-calling tool, we ran seven pipelines starting from the initial stage of taking input of raw fast5 files to the final output of the read-level and genome-level prediction results using the same high-performance computing (HPC) platform and environment for all seven tools (See “Methods”). We then split the raw ONT data to parallelize methylation calling for each tool. To minimize run time, a GPU and eight processors of hardware resources were allocated to each job for the methylation-calling tools. The SLURM resource and job management system effectively monitor the use of computing resources on HPC clusters [64]. Therefore, for each tested dataset we ran all jobs managed by SLURM and calculated the sums of CPU utilized times (hours), the max of job wall-clock times (hours), and the peak memory use (GB) based on reported logs of SLURM jobs for each pipeline (Fig. 7, Additional file 2: Table S12). We noted that only METEORE depends on the 5mC prediction of other tools’ results (e.g., Megalodon and DeepSignal), and thus the actual running time is the sum of the running times of METEORE, Megalodon, and DeepSignal. Guppy processed the fast5 raw signal file for NA19240 (~ 32× coverage) in the shortest amount of CPU time (151 h), followed by Megalodon and Nanopolish (698 and 703 h), while the CPU times for Tombo, DeepSignal, and DeepMod for the same file were much longer than the time required by Guppy (42×, 150×, and 186× longer, respectively). Furthermore, Guppy and Nanopolish exhibited the lowest peak memory usage (~ 13 and 19 GB), while Megalodon exhibited the highest peak memory usage (17× higher than that of Guppy). The same analysis of run time and peak memory usage for the other three benchmark datasets confirmed the ranking for these tools (Additional file 2: Table S12). In conclusion, Guppy and Nanopolish required both the least amount of CPU time and exhibited the lowest peak memory usage. DeepSignal and Tombo consumed more CPU times, but low peak memory, while Megalodon consumed large peak memory but short CPU time. METEORE and DeepMod both require the highest peak memory and CPU running time.

## Discussion

Robust detection of DNA methylation in the human genome is critical to improve our understanding of the functional impacts of epigenetic modifications. Recently, ONT nanopore-based sequencers have made possible direct DNA sequencing to generate long reads at single-molecule, single-base resolution. ONT long-read sequencing facilitates linkage of base modifications to genetic variants, and to specific regions of nucleic acids in the variants. Therefore, it allows exploration of epigenetic heterogeneity at single-molecule resolution and can improve our ability to detect long-range epigenetic phasing.

ONT has released multiple commercialized platforms and pore-chemistry versions (See timeline in Fig. 1A). In 2015, ONT released its first commercialized platform, MinION™ [65, 66], a portable device enabling simultaneous sequencing using up to 512 pores, with the capacity to generate up to 30 GB of DNA data [67]. In 2017, ONT introduced a scaled-up platform, GridION™, allowing analysis of up to five MinION flow cells and generation of up to 100 GB of data per run [16]. In 2018, ONT introduced the ultra-high-throughput platform PromethION™, with up to 48 flow cells [17], and later offered PromethION24/48 for much larger-scale sequencing [14]. Nanopore sequencing is considered a paradigm shift among recent sequencing approaches, because of its unique design enabling significant portability and relatively low cost [11, 68].

In the past, the advantages of long reads and real-time sequencing have made nanopore sequencing an effective tool to detect genomic and genetic aberrations such as DNA structural variants and RNA alternative splicing events [69]. Nanopore sequencing has demonstrated its powerful structural-variation-detection capacity in lung cancer [70, 71], leukemia [72], and neuron disorders [73–75], and it has been applied to clinical samples for molecular etiology or diagnosis of diseases associated with genomic variants [73–78]. Meanwhile, nanopore sequencing of splicing changes has been used research on cancers including breast cancer [79], leukemia [80, 81], and brain tumors [82]. Such research with nanopore sequencing has improved our understanding of evolutionary processes in human diseases. Furthermore, nanopore sequencing has opened new avenues for epigenetic research. For example, Miga et al. provide telomere-to-telomere assembly and DNA methylation maps of the human X chromosome [83] using nanopore sequencing, and Ewing et al. developed a long-read nanopore sequencing software for transposable elements (TE) detection and characterized the TE DNA methylation patterns [84]. Recently, efforts have been made to combine nanopore sequencing and other methods to perform epigenomics profiling and chromosome structure exploration. For example, Wongsurawat et al. used nanopore Cas9-targeted sequencing to simultaneously assess IDH mutation status and MGMT methylation levels in both cell lines and fresh biopsies of diffuse glioma [85]. Lee et al. developed a new nanopore sequencing-based method to simultaneously detect CpG methylation and chromatin accessibility [86]. Also, several preprint papers report the use of nanopore sequencing to enhance the understanding of epigenetic heterogeneity and the underlying molecular mechanisms [87–89].

Main advantages of nanopore sequencing include the ability to distinguish signals of 5hmC from that of 5mC and the allele-specific methylation (ASM). 5mC and 5hmC affect the electronic currents differently in the pore when DNA passes through: 5mC consistently increases the current relative to C, while 5hmC generally decreases the current relative to C, which reveals the potential feasibility to discriminate 5hmC, 5mC, and 5C by electric signal deviations [26, 30]. However, very few computational methods are available that can predict 5hmC from nanopore reads. SignalAlign [39]—a three-way (C, 5mC, or 5hmC) cytosine classifier trained by synthetic oligonucleotides—achieved an accuracy of 79% for predicting cytosine with 5hmC, but the method is developed with nanopore chemical version R7.3 (the pore is out-of-date and no longer available) and its repository has not been updated for over 4 years. We also noticed that ONT recently published a “research release” on basecalling model trained in 5hmC and 5mC in all contexts in the Rerio repository [90], and Megalodon [36] will be able to predict 5hmCs and 5mCs simultaneously. However, the 5hmC model is still under development, and to date no data are available on its performance to predict 5hmC. In the current study, we demonstrated that the ability to distinguish 5hmC from 5mC by nanopore sequencing, at least partly contribute to the discrepancy between BS-seq and nanopore sequencing, suggesting the great potential for simultaneous profiling of 5hmC and 5mC at single-base, single-molecule resolution by nanopore sequencing. Furthermore, nanopore sequencing is ideal to detect and phase ASM, considering the ability to detect multiple types of modifications (e.g., 5mC, 5hmC, 6 mA) from the same DNA molecule in a long range, For example, Akbari et al. [43] have developed a software to detect ASM in both human B-lymphocyte and B-lymphoblast cell lines using nanopore sequencing data. When more computational tools emerge for simultaneous prediction of multiple modifications (e.g., 5hmC and 5mC) from single read, these tools will likely expand the ability of allele-specific modifications detection by nanopore sequencing.

Previous benchmark work [38, 43] on methylation-calling tools for nanopore sequencing either compared a limited number of tools or evaluated limited CpG sites. Akbari et al. [43] focused primarily on methylation phasing using long reads and compared only three tools, using one publicly available nanopore sequencing dataset (R9.4). Also, only a few genomic contexts (genic regions and CpG islands) and per-site (i.e., single-base resolution) were included in the evaluation of methylation calling performance. Also, only a few genomic contexts (genic regions and CpG islands) and per site. Yuen et al. [38] performed benchmarking evaluation on selected 100 CpGs from *E. coli* and 1743 CpGs from human genome using NA12878 Cas9-targeted nanopore sequencing. Our current work is distinctive from the prior studies. First, we presented a systematic benchmark of all seven current available methylation-calling tools for nanopore sequencing data generated from human natural DNA at the whole-genome scale, not using a limited number of CpG sites or a limited number of tools. A whole human-genome scale comparison is critical. For example, while we confirmed that the performance of DeepMod was comparable to that of other tools for the bacteria genome, DeepMod performed poorly at the human whole-genome scale, which was not previously reported. Also, we showed that the performance of METEORE across the human genome was worse than that of Nanopolish, Megalodon, DeepSignal, and Guppy at the genome-wide scale across four datasets, which is distinctive from the evaluation by Yuen et al. [38]. This is likely due to their small training dataset—100 CpG sites from *E. coli*—and the performance of METEORE was evaluated based on less than 2000 CpG sites. Second, to compliment current publicly available benchmark datasets generated from human cell lines, we generated (1) two new nanopore sequencing datasets: one is from a primary leukemia specimen, and one is a human leukemia cancer cell line, and (2) WGBS and oxBS-seq datasets. The new datasets enable the evaluation of these tools in a primary human specimen, not only in human cell lines, and thus will provide guidance on the application of nanopore sequencing in clinical research. In total, we used four human datasets with different coverages, which are large benchmark datasets than the prior benchmark studies. Third, we evaluated the prediction robustness not only at a per-site level, but also at a per-read level, and considered more diverse genomic contexts, e.g., singletons, discordant and concordant non-singletons, genic and intergenic regions, various CG density regions, repetitive regions, and CTCF binding regions. Fourth, we demonstrated that the 5hmC levels contribute to the discrepancy between BS-seq and nanopore sequencing. Fifth, we also compared the number of CpGs predicted by each tool and the computational resources consumed by each tool. For example, the raw fast5 data from a single nanopore sequencing library, e.g., NA12878, with ~ 26× coverage, can use over 30 TB of storage space and over hundreds to thousands of CPU hours. Thus, the consumption of computational resources is essential for guiding the design of data analyses on HPC and cloud computing platforms for large-scale human nanopore sequencing data.

## Conclusion

Oxford Nanopore long-read sequencing technology poses both opportunities and challenges for accurate methylation prediction and long-range epigenetic phasing. The past few years have witnessed rapid development of both the sequencing technology and analytical tools. For DNA methylation analysis, many algorithms are emerging for nanopore sequencing data, and we comprehensively surveyed all current publicly available computational tools.

**Fig. 8.**
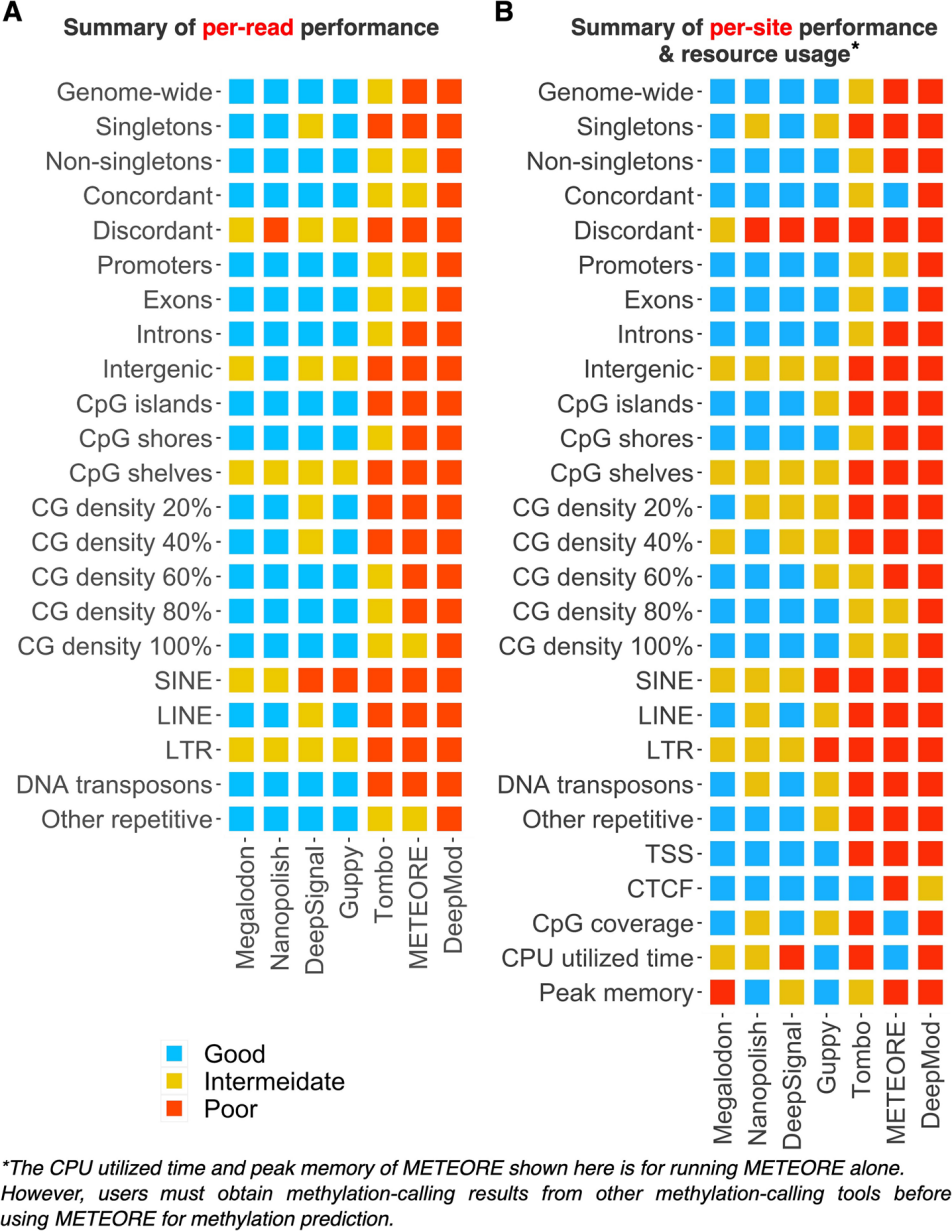
Summary of per-read and per-site performances across all major evaluation criteria. Summary of **A** per-read performance (F1 score), **B** per-site performance (Pearson correlation coefficient), and resource usage. Tools are ranked by their average performance across the criteria, with the numerical values of good = 2, intermediate = 1, poor = 0. Details in evaluation criteria and cutoff values for performance categories are available in the Methods and Additional file 2: Table S13

Based on our systematic comparison, we summarized the performances of seven tools across all major evaluation criteria (Fig. 8). For each evaluation criterion, the tools were classified as “good,” “intermediate,” or “poor” (in “Methods” and Additional file 2: Table S13). We derived five key observations. First, the choice of methylation-calling tool critically affects the level of the F1 score, accuracy, and the AUC score at different genomic regions. Overall, the top performers were Megalodon, Nanopolish, DeepSignal, and Guppy. The consensus approach METEORE integrating other tools was reported to have better performance than individual tools in 100 CpG sites of *E. coli* genome and 10 regions from human genome, but at human-genome scale it was not as good as the top four performers. DeepMod exhibited comparable performance for *E. coli* genome but not in human genome in our benchmark analysis. Second, detection of 5mCs at regions with discordant DNA methylation patterns, intergenic regions, low CG density regions, and repetitive regions (i.e., SINE and LTR) showed room for improvement across all tools. Therefore, penalized models, i.e., imposing an additional cost on the models for making classification mistakes at these regions, or expand the training datasets on these more challenging regions, may enhance the robustness of methylation calling for these biologically interesting regions. Third, Guppy and Nanopolish had the lowest memory usage, while Guppy, Nanopolish, and Megalodon are faster than all other tools. Fourth, we confirmed that the discrepancy in 5mC levels between the BS-seq and nanopore sequencing data results in part from the 5hmC modifications. Unlike nanopore sequencing, BS-seq cannot distinguish between 5mCs and 5hmCs, as bisulfite treatment does not convert either modification. Fifth, Nanopolish and Guppy are fast, but they detected, respectively, 6 and 4% fewer CpG sites than did DeepSignal and Megalodon, due to the more stringent log-likelihood ratio cutoff used by Nanopolish and Guppy for predicting non-singleton CpG sites. Thus, methylation calling using nanopore sequencing will benefit from future endeavors to increase the accuracy of challenging regions, the predicted CpG coverage, and high efficiency [91] in using computing resources.

Therefore, we believe that our benchmarking of methylation-calling tools will guide researchers in making well-considered and effective choices when designing an analytic plan for epigenomic profiling using ONT sequencing, including Cas9-targeted nanopore sequencing data analysis. For users with limited computational resources, we recommend Guppy and Nanopolish for methylation analysis. Guppy requires minimum CPU hours and peak memory as one of the top four performers because the base-modification prediction is part of its basecalling. Nanopolish is the best option considering per-read and per-site performance criteria, as well as low CPU hours and peak memory usage after basecalling. For users with the access to HPC resources or a larger budget for cloud computing resources, Megalodon is the best option, considering its performance in the more challenging areas including repetitive regions and discordant non-singletons, also as it predicts more CpG sites compared to Nanopolish and Guppy. Robust prediction of DNA methylation at different genomic contexts will help improve our understanding of epigenetic mechanisms in gene regulation underlying many biological processes, including mammalian normal development, aging, and complex disease development.

## Methods

### Sample collection and processing

In the study, we used four independent human datasets: two normal B-lymphocyte cell lines (NA19240 [56], NA12878 [57]), one primary acute promyelocytic leukemia clinical specimen (APL), and one cancer cell line (K562).

The APL sample was obtained from the Stem Cell and Xenograft Core of the University of Pennsylvania. The Core maintains a tissue bank of cells from patients with hematologic malignancies. This is Institutional Review Board (IRB)-approved research (IRB protocol #703185). The patient sample was collected at the time of clinical presentation and prior to therapy. The sample was collected as leukapheresis and viably frozen using standard techniques. The de-identified specimen was then provided to the Jackson Laboratory for Genomic Medicine (JAX-GM). Diagnosis of APL was confirmed by fluorescence in situ hybridization (FISH) analysis for *t*(15;17).

K562 was cultivated in Roswell Park Memorial Institute (RPMI) 1640 Medium (Gibco, A10491-01) with 10% fetal bovine serum (FBS) (Gibco, 26140079). K562 medium was additionally supplemented with 1% Antibiotic-Antimycotic (Gibco, 15240062).

### Genome annotations download and preprocessing

We downloaded the following genome annotation files from the UCSC Genome Browser: GRCh38/hg38 chromosome sizes; GRCh37/hg19 chromosome sizes; RepeatMarker annotations for hg38; hg38 CpG islands locations (cpgIslandExt.txt); hg38 GC percent in 5-base windows (hg38.gc5Base.wigVarStep.gz). We downloaded the v26 comprehensive genes annotation file (gencode.v26.annotation.gtf.gz) from GENCODE.

All analyses were restricted to chromosomes 1-22, X, and Y. Promoters were generated by extending the region 2000 bp up and down from the TSS. Genes were subtracted with the “gene” feature type, while exons were subtracted with the “exon” feature type from the GENCODE v26 annotation genes file. Introns were generated by taking the difference between the genes file and the exons file. Intergenic regions were generated by taking the difference between the reference genome and all other gene feature types (gene, CDS, promoter, intron) from the gene annotation file using *bedtools subtract*. CpG shores were generated by extending the region 2 kb up and down from the hg38 CpG island location file and were subtracted the overlapped CpG islands, while CpG shelves were generated from 2 to 4 kb from CpG islands and were subtracted the overlapped CpG islands and shores. DNA repetitive regions were restricted to classes SINE, LINE, LTR, DNA transposons, and others (i.e., all classes other than SINE, LINE, LTR, and DNA transposons were combined into a single category named “Others”).

### BS-seq datasets and analysis

We generated WGBS and oxBS-seq for APL. DNA was extracted using the AllPrep DNA/ RNA kit (Qiagen) following the manufacturer’s recommendation. Two 500 ng fragments of DNA were sheared to 500 bp using a LE220 focused-ultrasonicator (Covaris) and purified using 0.9X SPRI beads (Beckman Coulter). The libraries were prepared using the KAPA Hyper Prep Kit for Illumina (Roche), and bisulfite conversion was performed using the TrueMethyl Seq Kit (CEGX). Briefly, the fragmented DNA was first spiked in with CEGX sequencing controls, followed by end-repair and A-tailing, and then ligated with a SeqCap indexed adaptor (Roche). The sample destined for oxBS-seq was first subjected to oxidation whereas the sample destined for the WGBS library were mock-treated, and then followed by a bisulfite conversion. After purification, the bisulfite-treated DNA was amplified with 15 cycles of PCR. The final library was quantified by real-time qPCR for an accurate concentration since proper quantitation is needed for loading the library for next-generation sequencing. Libraries were sequenced paired-end 2 × 150 bp on an Illumina HiSeq 2500 instrument.

We used the published WGBS data for K562 and NA12878, and the published RRBS data for NA19240. The BS-seq data for NA19240 and APL were analyzed with Bismark [92] with the human reference genome (GRCh38/hg38) to obtain the cytosine methylation frequency at each CpG site. Region-specific analysis and local smoothing for samples was performed using our in-house BS-seq pipeline (https://github.com/TheJacksonLaboratory/BS-seq-pipleine). For NA12878 and K562, we obtained the BED file directly from ENCODE. Specially, we took the union of CpG sites from two replicates (WGBS or RRBS) as corresponding DNA methylation ground truth. Then, we selected CpG sites with coverage ≥ 5 for per-read performance of 5mC prediction evaluation, where a CpG is either fully methylated with 100% methylation frequency is or unmethylated with zero methylation frequency. In total, for WGBS, 15,371,020 high-confidence CpG sites were selected for NA12878; 25,382,453 sites for K562, and 8,707,604 sites for APL. For RRBS, 251,145 sites were selected for NA19240 (Additional file 2: Table S2). For each dataset, we took the intersections of fully methylated or unmethylated CpG sites from BS-seq and those from all tools for per-read performance evaluation.

We used the statistical method MLML [60] to simultaneously estimate 5mC and 5hmC levels at each CpG site from paired WGBS and oxBS-seq (coverage ≥ 1). Only intersected CpG sites in both WGBS and oxBS-seq with zero conflict were considered for further analysis. Specially, if the estimated methylation level falls out of the confidence interval of binomial test calculated from input coverage and methylation level, then such event is counted as one conflict; the site is not reliable if more conflicts happen on one site [93].

### Nanopore sequencing datasets

We generated nanopore sequencing datasets for APL and K562 at JAX-GM. For APL and K562, genomic libraries were prepared using the Rapid Sequencing Kit (SQK-RAD004, ONT) according to the manufacturer’s recommendation. Briefly, 1200 ng DNA was incubated with 2.5 μl of FRA at 30 °C for 1 min and 80 °C for 1 min. This was followed by the addition of 3 μl of adaptor (RAP) to the reaction mix, and the mixture was then incubated at 5 min at room temperature. The libraries were sequenced on Flow Cell R9.4.1 (FLO-MIN106, ONT) on GridION (ONT) using MinKNOW software for 48 h.

NA19240 was sequenced for the 1000 Genomes Project [56] and the sequencing depth was ~ 32× coverage (the total number of bases sequenced divided by the total number of genome bases). We obtained nanopore raw data and their library preparation details from the authors of [56]. HMW genomic DNA was extracted using the phenol chloroform approach [94]. Libraries were prepared using the 1D Ligation Sequencing Kit (SQK-LSK108, ONT) according to the manufacturer’s recommendation. The library was sequenced on Flow Cell R9.4.1 (FLO-MIN106, ONT) on a GridION (ONT) using the MinKNOW software for 48 h.

NA12878 was sequenced with a reported median 26× coverage by the Whole Human Genome Sequencing Project [57]. The NA12878 human genome was sequenced on the ONT MinION with R9.4 chemistry (FLO-MIN106) using the 1D Ligation Sequencing Kit (SQK-LSK108, ONT). We downloaded nanopore raw data from the GitHub repository (https://github.com/nanopore-wgs-consortium/NA12878). We downloaded the *E. coli* positive control dataset generated by Simpson et al. [9] on GitHub (https://github.com/comprna/METEORE), which contains 50 single-read fast5 files.

### Nanopore sequencing data preprocessing

Basecalling, the process of translating raw electrical signal of nanopore sequencing into nucleotide sequence, is the initial step of nanopore data analysis. Both ONT and independent researchers are actively developing different tools for the basecalling step. Specifically, ONT provides basecalling programs including official ONT community-only software (Albacore and Guppy) and open-source software (Flappie, Scrappie, Taiyaki, Runnie, and Bonito [95]), the latter of which are under development with new algorithms for basecalling. Among the basecalling programs, Albacore and Guppy are compatible with Oxford Nanopore R9.4 reads and offer the most stable performance [54]. Albacore [96] is a general-purpose base caller that runs on CPUs. Guppy [51] is a neural network based basecaller with several bioinformatic post-processing features. Guppy supports both CPUs and GPUs for improved basecalling run time, and it is available on the ONT community site (https://community.nanoporetech.com). ONT discontinued to develop Albacore due to the better performance of Guppy [54]. Because the state-of-art basecaller Guppy using the default high-accuracy (HAC) model showed excellent performance among ONT basecalling tools [54], we used Guppy (v4.2.2) with default model (dna_r9.4.1_450bps_hac.cfg) for basecalling for all nanopore reads (fast5 files). The basecalled reads were then aligned to the human reference genome (GRCh38/hg38) for human datasets or aligned to the *E. coli* K12 MG1655 genome for the *E. coli* dataset using minimap2 [53]. Specially, R9.4-series pore is the current broadly used ONT flow cell and there is a slight difference between R9.4 and R9.4.1 flow cells, and all the models used in our work can work for both [97]. Moreover, for cell line authentication of K562, we aligned the basecalled reads to the human reference genome (GRCh37/hg19) using minimap2 [53] and Samtools [98] and compared the aligned reads at the target regions with reported insertions/deletions (indels) derived from the Cancer Cell Line Encyclopedia (CCLE) project [99] in genome browser IGV.

### Methylation-calling tools for nanopore sequencing

We evaluated the performance of Nanopolish (v0.13.2), Megalodon (v2.2.9), DeepSignal (v0.1.8), Guppy (v4.2.2), Tombo (v1.5.1), METEORE (v1.0.0), and DeepMod (v0.1.3) to detect 5mCs. These seven tools differ in the underlying algorithms and the modifications they are trained to detect DNA methylation.

Nanopolish [9] calls 5mCs in a CpG context using a HMM to assign a log-likelihood ratio (LLR) for each CpG site, where a positive log-likelihood ratio (LLR) indicates support for methylation. Nanopolish groups nearby CpG sites together and calls the cluster jointly to assign the same methylation status to each site in the group. For example, on a motif such as CGCGT, Nanopolish reports a LLR for the whole group, rather than a separate LLR for the individual cytosine. We used 2.0 as the LLR threshold for methylation calling, as the Nanopolish authors suggest on the GitHub that the initial 2.5 shown in the paper is overly conservative, and the default threshold was replaced with 2.0 from v0.12.0 [63]. Specifically, we first detected methylated CpGs (LLR > 2.0) and unmethylated CpGs (LLR < -2.0) at the read level and removed ambiguous predictions (− 2.0 ≤ LLR ≤ 2.0). Then we calculated per-site methylation frequency by the fraction of reads classified as methylated.

Megalodon [36] is a new ONT-developed command line tool that can identify modified base using deep learning recurrent neural network (RNN) model by utilizing Guppy (v ≥ 4.0) pre-trained models for basecalling on the backend. Megalodon predict 5mC at either the per-read or per-site level (by aggregating per-read results) based on the log probability that the base is modified or unmodified. Guppy (v ≥ 4.0) backend and pre-trained models is recommended for basecalling [36], and therefore, we fed Megalodon v2.2.9 with Guppy v4.2.2 with the latest “5mC in an all context” model (res_dna_r941_min_modbases_5mC_v001.cfg) from Rerio [90], and chose the default probability cutoff (0.8) to predict DNA methylation.

DeepSignal [35] proposed a RNN with a Bidirectional Long/Short-Term Memory (BiLSTM) and Inception structure to detect the methylation state of target cytosine in a CpG context. DeepSignal required an extra re-squiggle module of Tombo before methylation calling. The methylation calling output of DeepSignal is a tab-delimited text file at read level including two probability values for each base, one for methylated (prob_1) and one for unmethylated (prob_0), as well as a binary call, i.e., unmethylated (prob_1 > prob_0) or methylated (prob_1 ≤ prob_0) for each base. We predicted per-read 5mC with the CpG model trained using HX1 R9.4 1D reads (*model.CpG.R9.4_1D.human_hx1.bn17.sn360.v0.1.7 + .tar.gz*) provided by DeepSignal from the GitHub repository at https://github.com/bioinfomaticsCSU/deepsignal and calculated the per-site 5mC level using their official methylation frequency script.

Guppy is a ONT-developed basecaller [51] and is able to identify certain types of modified basecalling (i.e., 5mC, 6 mA) from the raw signal data [32]. We first used a Guppy methylation-calling model (dna_r9.4.1_450bps_modbases_dam-dcm-cpg_hac.cfg) to estimate modified base probabilities with integer scores in the range [0, 256]. Then, we used ONT-developed fast5mod (v1.0.5) [55] default score cutoffs to convert Guppy’s modified base probabilities into modified base predictions, and kept the predictions in a CpG context. Specifically, fast5mod output per-site 5mC level by the number of methylated (score > 128) reads were divided by the sum of methylated reads and unmethylated (score < 64) reads.

ONT-developed Tombo [20] performed a statistical test to identify modified nucleotides without the need for the training data. Tombo computed per-read, per-site test statistics by comparing the signal intensity difference between modified bases and unmodified bases. We chose to use the recommended CpG motif-specific model with the default threshold of (− 1.5, 2.5) for DNA where scores below − 1.5 were considered as methylated and above 2.5 unmethylated, and scores between these thresholds did not contribute to the per-site methylation.

DeepMod [34] designed a bidirectional RNN with an LSTM unit for genome-scale detection of DNA modifications. The input is a reference genome and fast5 files with raw signals basecalled by Guppy (v4.2.2). The output is a BED file with coverage, number of methylated reads, and methylation percentage information for genomic positions of interest. Since a 5mCs in a CpG motif has a cluster effect in the human genome [34], DeepMod provides a cluster model to generate a final output for site-level-predicted methylation probability in the human genome. We performed DeepMod for methylation calling with the RNN model (rnn_conmodC_P100wd21_f7ne1u0_4) and cluster model (na12878_cluster_train_mod-keep_prob0.7-nb25-chr1) from the GitHub repository at https://github.com/WGLab/DeepMod. Also, since DeepMod aggregated methylation calling results into a per-site output BED file, we counted the number of methylated callings and unmethylated callings from BED outputs to evaluate its read-level performance.

METEORE [38] is a consensus approach combining the predictions from two or more methylation-calling tools. Currently, neither METEORE regression pre-trained models nor training datasets for regression models are available. METEORE random forest (RF) model combining Megalodon and DeepSignal achieved lower root mean square error (RMSE) than other available METEORE models [38]. Thus, we used the METEORE pre-trained RF model to combine the outputs from Megalodon and DeepSignal to predict methylation state at per-site level with a methylation probability threshold of score 0.5, i.e., the site is called as unmethylated when the score ≤ 0.5, and the site is called as methylated when the score > 0.5. After that, we used METEORE script to calculate the methylation frequency at a site level.

The performances of these methods that use prior knowledge about the expected deviations in signal are highly dependent on the training data, which is typically composed of a fully unmodified and a fully modified sample. Motifs that are not represented in the training set or that contain mixtures of modified and unmodified bases may lead to suboptimal performance.

### Per-read performance evaluation

We designed the performance-evaluation process for 5mC predictions among seven tools as follows. First, we identified those CpG sites shared by in BS-seq (coverage ≥ 5) and predicted each nanopore sequencing methylation-calling tools (coverage ≥ 1). We only kept CpG sites that showed 0 or 100% methylation levels by BS-seq (ground truth), as we need to evaluate the per-read performance of these tools as classification models. Second, we calculated the F1 score, accuracy, precision, and recall and assessed the tradeoff between true-positive and false-positive rates of 5mC predictions by calculating the ROC curve by varying the threshold for methylation calling and reported the AUC values as follows:

- Precision = TP/(TP + FP)
- Recall = TP/(TP + FN)
- Accuracy = (TP + TN) / (TP + TN + FP + FN)
- F1 Score = 2 × (Recall × Precision) / (Recall + Precision)

Here, TP means true positive, TN means true negative, FP means false positive, and FN means false negative. We calculated F1 score for both 5mCs and 5Cs and used macro F1 score, i.e., average F1 score of 5mC and 5C, as the overall F1 score for each tool. AUC is a performance metric used to evaluate how well a classifier performs on both methylated and unmethylated class predictions.

### Per-site performance evaluation

First, we calculated Pearson correlation coefficients of methylation percentage for each pair of tools (coverage ≥ 3) and between each tool and BS-seq (coverage ≥ 5) at whole-genome level. Second, we computed the relationship between CpG methylation percentage with distance to TSS (bin size = 50 or 200 bp) and CTCF binding sites (bin size = 100, 125, or 200 bp) using deepTools [100].

### Memory usage and running time evaluation

We compared the memory usage and running time of the seven tools on the single-read fast5 files of each dataset. All tools have support for multi-processors, and we compare the scalability of these tools on the same system configurations. We split the ONT datasets for parallelization. All tools were carried out on the same computer clusters with the following configurations: 32 cores; 300 GB RAM; 1 TB Data Direct Networks Gridscalar GS7k GPFS storage appliance. The HPC platform software and hardware specifications are as follows: slurm manager version: 19.05.5, CPU: Intel(R) Xeon(R) Gold 6136 CPU @ 3.00 GHz, GPU: Tesla V100-SXM2-32GB. Each job was allocated eight processors, 300 GB memory, and one GPU hardware resource (GPU was allocated for running Guppy, Megalodon, DeepSignal, and DeepMod). We extract running time (field name: CPU Utilized), job wall-clock time (field name: Job Wall-clock time), and peak memory utilization (field name: Memory Utilized) from the SLURM job log data. These results were used as the measurement of running time and memory usage for hardware performance comparison and evaluation.

### Nanome web application implementation

To facilitate the dissemination of DNA methylation calling results using nanopore sequencing from the current benchmark study, we present a web application named nanome. Nanome is a user-friendly interactive nanopore sequencing methylation database and is implemented with Shiny package from R programming language. The database allows the users to select their features of interest, including chromosomes, strands, datasets, singletons and non-singletons, genomic contexts, regions of various CG density, and repeat regions. Nanome also provides methylation percentage and read coverage at each genome site across different methylation calling tools and bisulfite sequencing. Nanome is available as a hosted web application that runs within a web browser and can be accessed by https://nanome.jax.org.

### Performance summary criteria

Figure 8 summarized the performance of each methylation-calling tool across the range of evaluation metrics. We calculated the F1 scores, Pearson correlation coefficients, CPU utilized time, and peak memory by the median value of each tool achieved across the four datasets. The mean normalized CpG coverage (the number of CpGs divided by the number of the union of CpGs of all tools) was calculated for the high-coverage datasets NA19240 and NA12878. The performance for TSS and CTCF binding peaks was measured by the sum of the absolute difference between WGBS and each tool for NA12878. For each metric, the performance of each tool was considered either “good,” “intermediate,” or “poor” (Additional file 2: Table S13).

## Supplementary Information

**Additional file 1.** Contains supplementary figures S1-S8.

**Additional file 2.** Contains supplementary tables S1-S13.

**Additional file 3.** Review history.

## Data Availability

Nanome source codes are available at the GitHub repository (https://github.com/TheJacksonLaboratory/nanome) [101] and are deposited in Zenodo as DOI-assigned repository [102] under MIT license. WGBS and RRBS files are downloaded from the ENCODE portal [103] (https://www.encodeproject.org) with the following identifiers: ENCFF721JMB and ENCFF867JRG (WGBS for K562), ENCFF835NTC and ENCFF279HCL (WGBS for NA12878), ENCFF000LZS and ENCFF000LZT (RRBS for NA19240). ONT sequencing data for NA19240 is available upon request from Chaisson et al. [56]. ONT sequencing data for NA12878 from Jain et al. [57] is available as an Amazon Web Services Open Data Set at https://github.com/nanopore-wgs-consortium/NA12878. ONT sequencing data for *E. coli* dataset is from Yuen et al. [38], which is publicly available as a GitHub repository at https://github.com/comprna/METEORE. ONT sequencing data for K562 is deposited at the Gene Expression Omnibus (GEO) under the accession number GSE173688 [104]. WGBS, oxBS-seq, and ONT sequencing data for APL are deposited at the European Genome-phenome Archive (EGA) under accession number EGAS00001005610 (WGBS and oxBS-seq for APL) [105], and EGAS00001005613 (ONT for APL) [106].
