# Supplementary material for: DNA methylation-calling tools for Oxford Nanopore sequencing: a survey and human epigenome-wide evaluation
Source: Genome Biol. 2021 Oct 18;22:295. doi: 10.1186/s13059-021-02510-z (PMC8524990; doi:10.1186/s13059-021-02510-z)
Supplement: Supplementary file 1 — Additional file 1. Contains supplementary figures S1-S8. [file 13059_2021_2510_MOESM1_ESM.docx]

**Supplementary Figures**

**
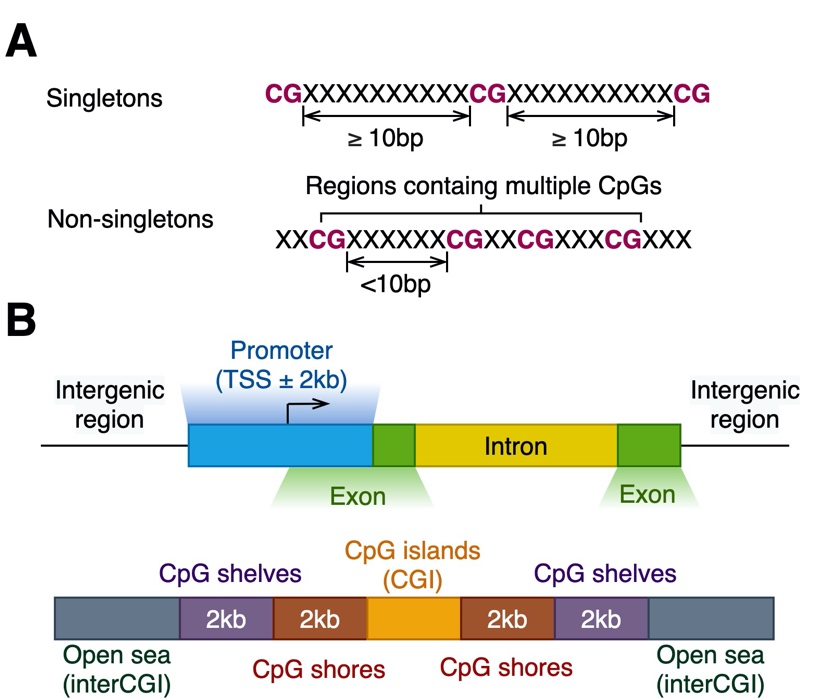
**

**Figure S1. Scheme for (A) singletons/non-singletons and (B) genic and intergenic regions, CpG islands, shores, and shelves.** Singletons are CpG sites with only one CpG up and down 10-base-pair regions. Non-singletons are CpG sites with multiple CpG sites up and down 10-base-pair regions.

**
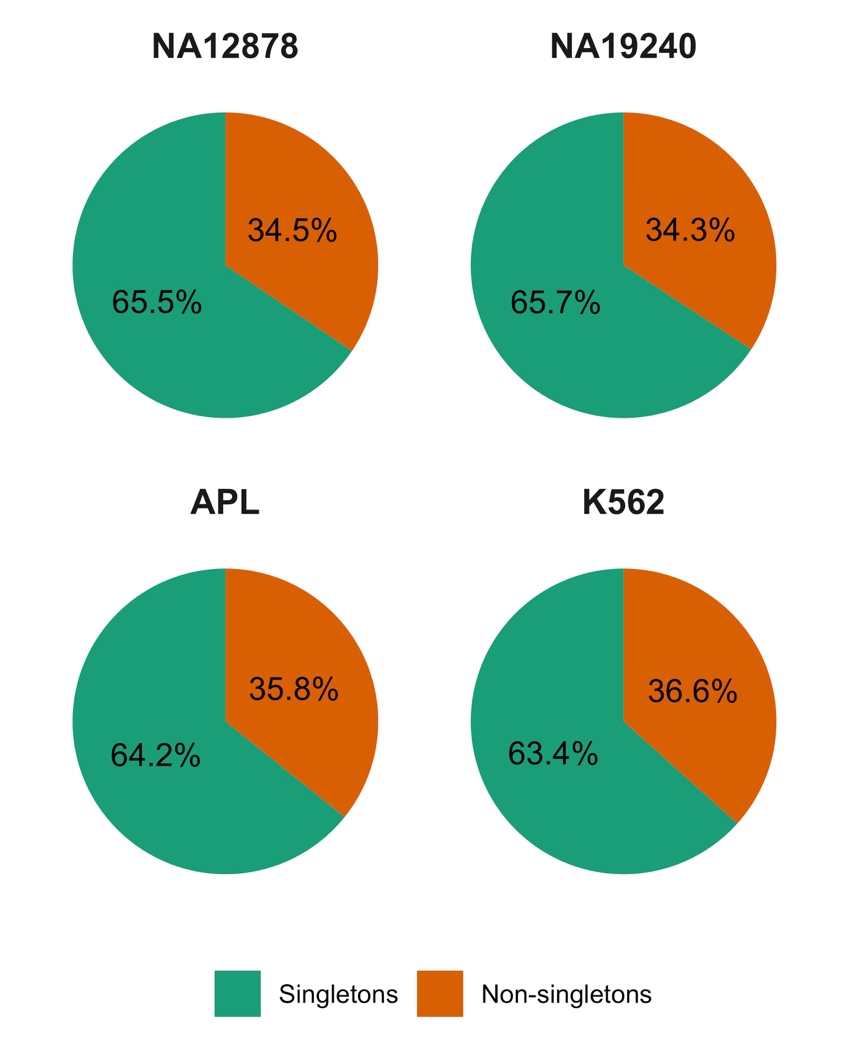
**

**Figure S2. Distribution of CpG sites at singleton and non-singleton regions covered by raw nanopore sequencing reads (read coverage≥3) for four datasets.** Singletons are CpG sites with only one CpG up and down 10-base-pair regions. Non-singletons are CpG sites with multiple CpG sites up and down 10-base-pair regions.

**A**

**
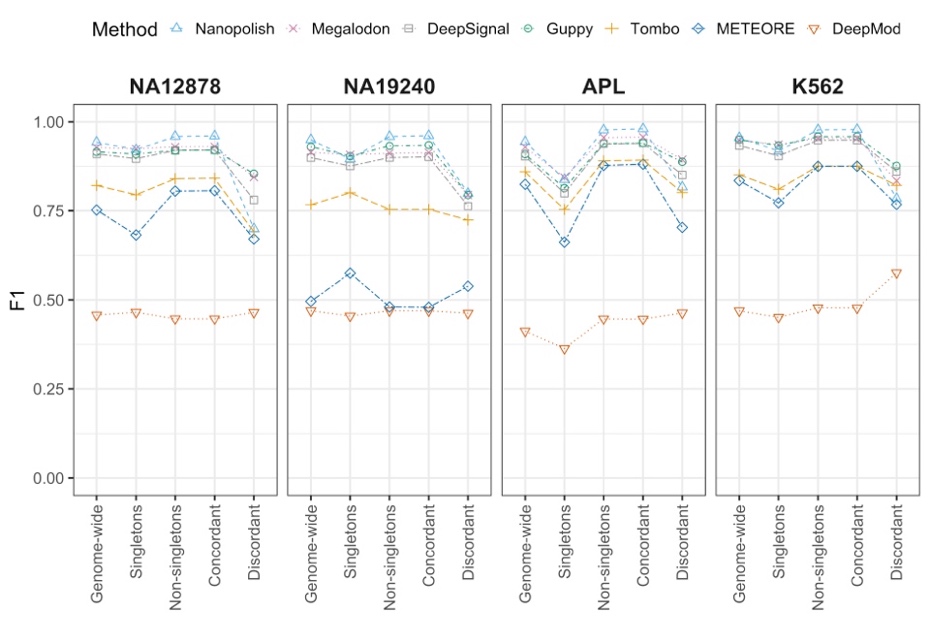
**

**B**

**
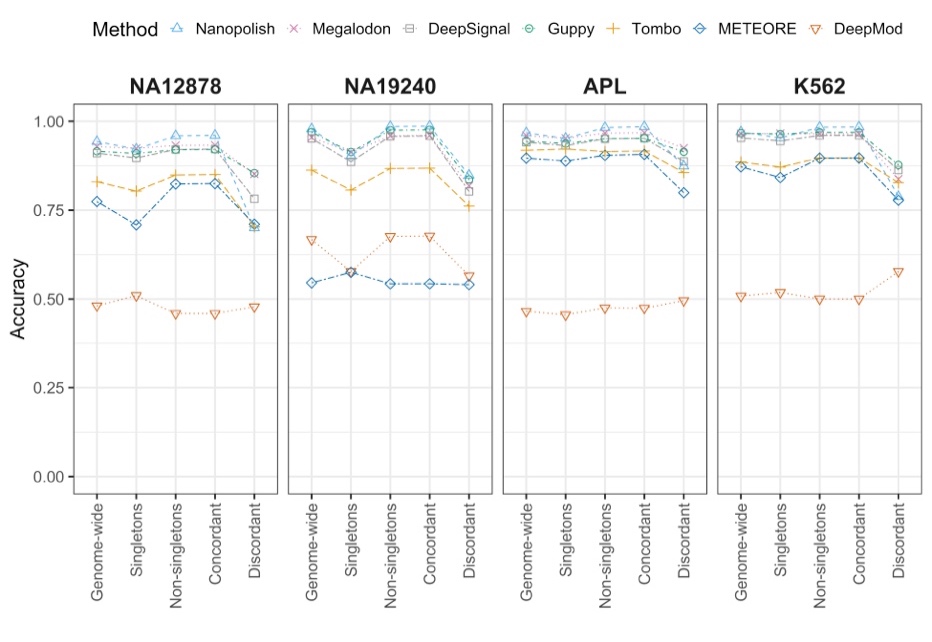
**

**C**

**
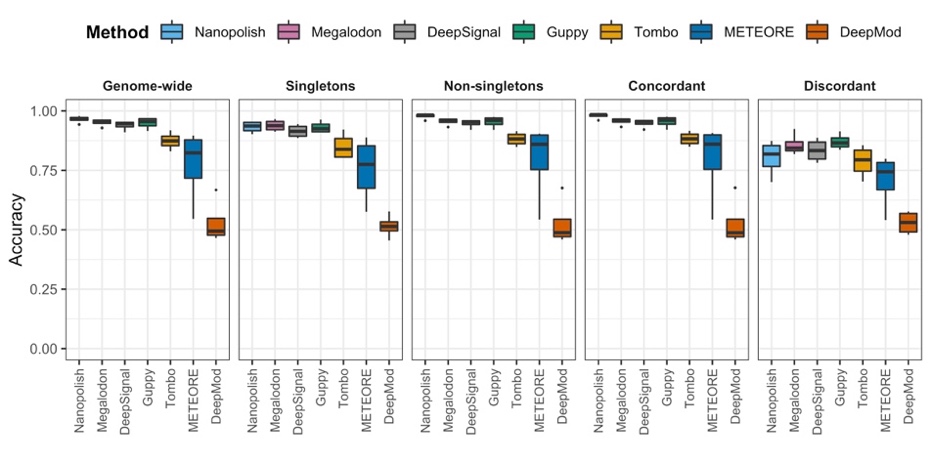
**

**Figure S3. Per-read performance (i.e., F1 score and accuracy) of 5mC prediction at singletons and non-singletons.** (**A**) F1 score and (**B**-**C**) accuracy achieved by all seven tools across four datasets. (**A-C**) BS-seq datasets served as ground truth for the evaluation.

**A**

**
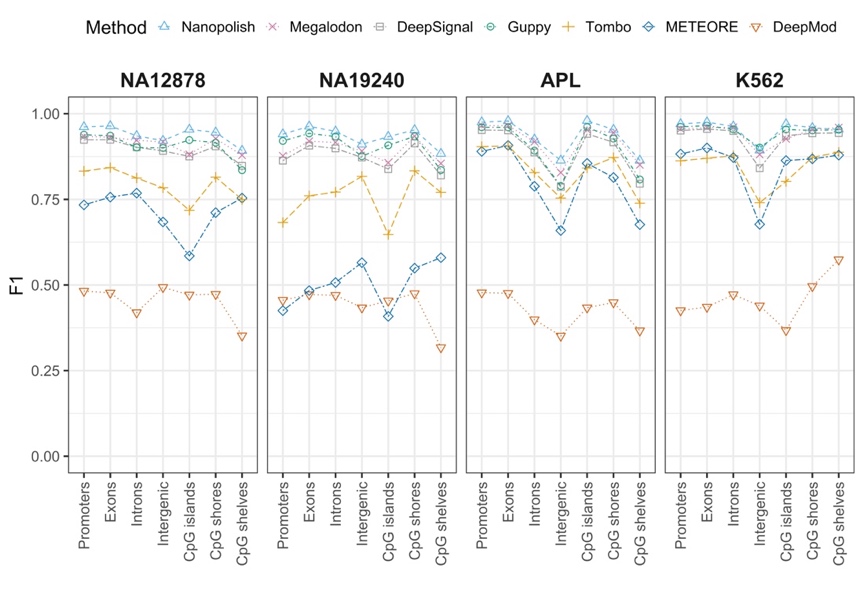
**

**B**

**
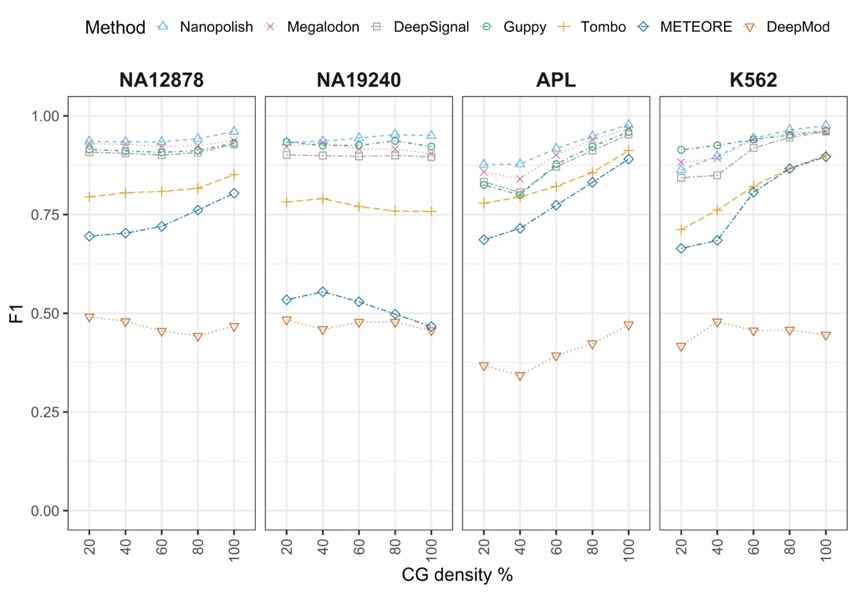
**

**C**

**
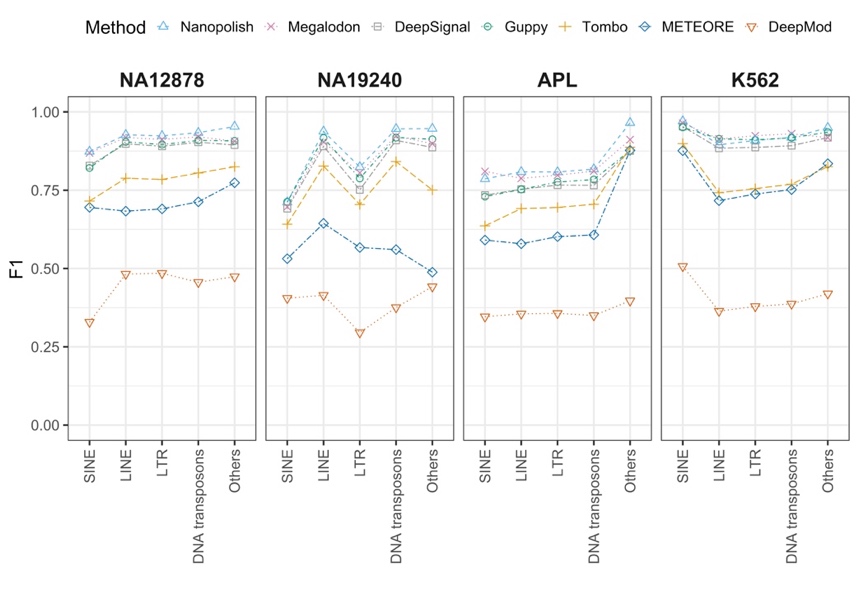
**

**Figure S4. Per-read performance of 5mC prediction across different genomic regions.** F1 score at (**A**) genic and intergenic regions, CpG islands, shores, and shelves, (**B**) regions of different CG densities, and (**C**) repetitive regions across four datasets. (**C**) We considered short interspersed nuclear elements (SINE), long interspersed nuclear elements (LINE), long terminal repeats (LTR), DNA transposons, and “Others” for other repetitive regions. (**A-C**) BS-seq datasets served as ground truth for the evaluation.

**A**

**
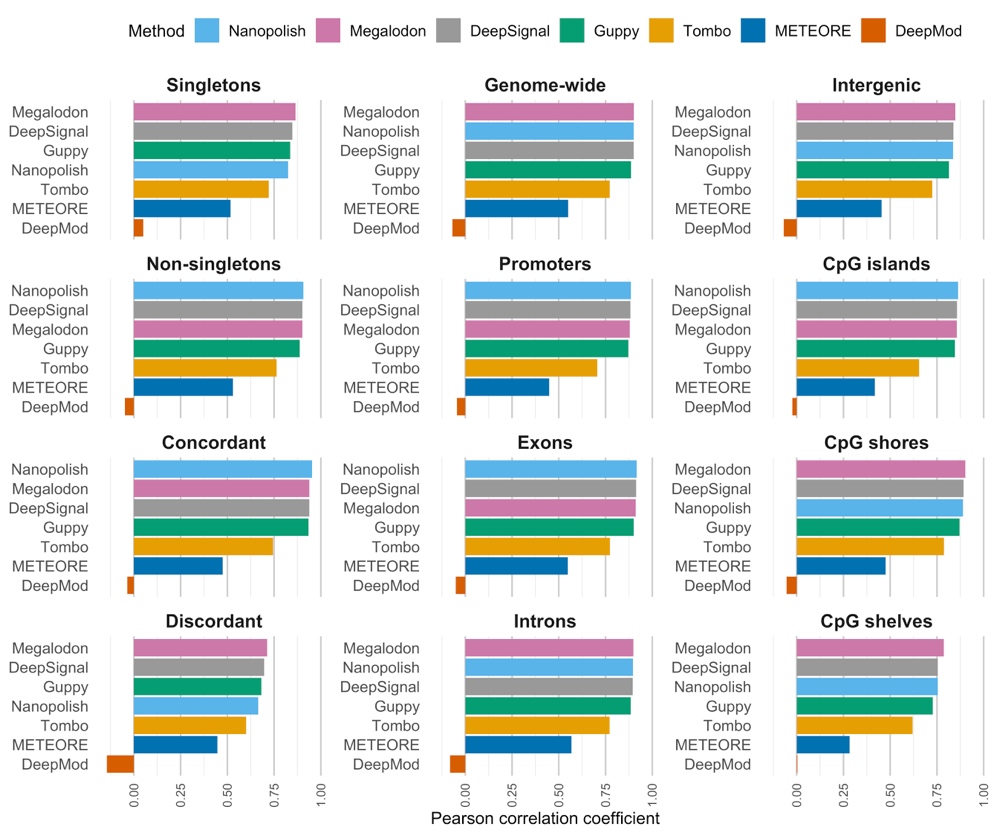
**

**B**

**
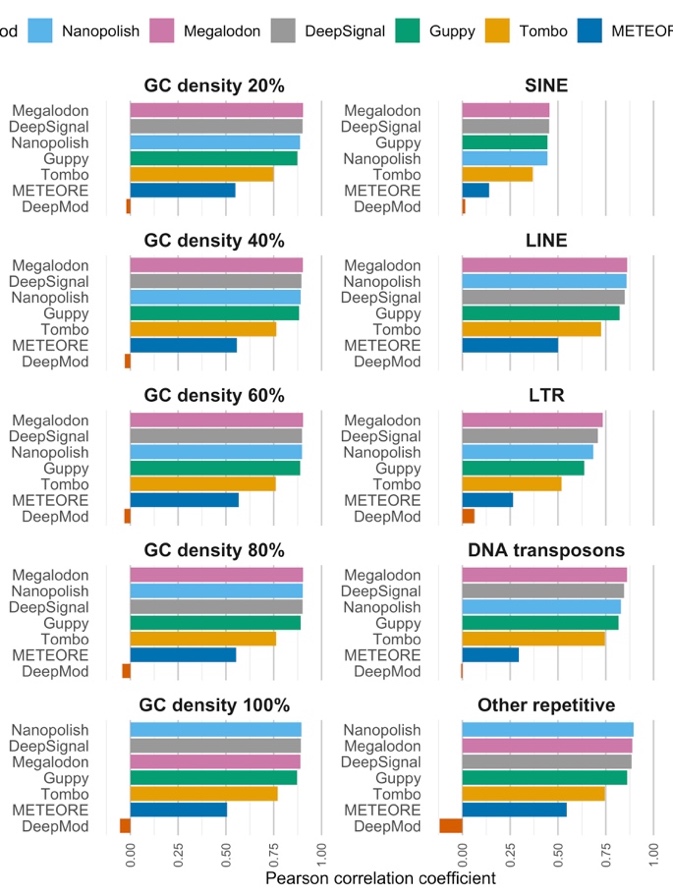
**

**Figure S5. (A-B) Per-site performance of 5mC prediction in different genomic contexts.** We used Pearson correlation coefficient between nanopore sequencing and BS-seq to evaluate per-site performance. We considered (**A**) singletons, non-singletons, genic and intergenic regions, CpG islands, shores, and shelves, (**B**) regions of different CG densities, and repetitive regions using the NA19240 dataset**.** The x-axis is the Pearson correlation coefficient, the y-axis represents each tool. The methylation-calling tools in the bar plots are sorted by Pearson correlation coefficient in descending order from top to bottom.

**
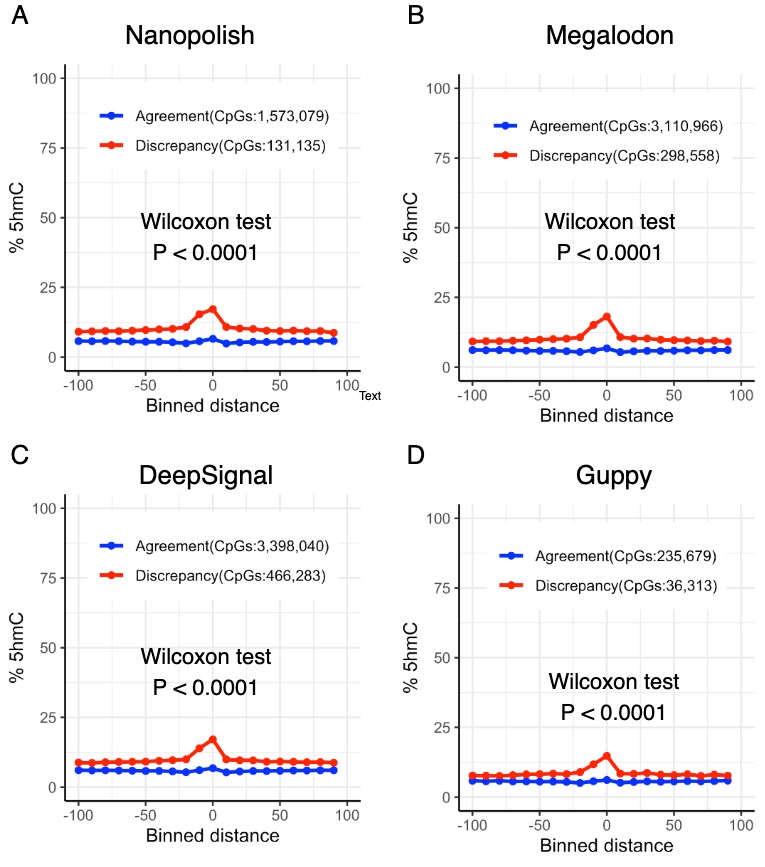
**

**Figure S6. Relationship between 5hmC percentage and distance to CpGs exhibiting agreement or discrepancy between nanopore sequencing and BS-seq.** We computed site-level 5mC percentage from APL nanopore sequencing data using (**A**) Nanopolish, (**B**) Megalodon, (**C**) DeepSignal and (**D**) Guppy. The CpGs exhibiting agreement are sites where the 5mC differences between Nanopolish and BS-seq are less than 5%, and the CpGs exhibiting discrepancy are sites where the 5mC differences are greater than 40%. The Wilcoxon rank sum test was analyzed and reported for the center bin (binned distance = 0). The bin size is 10 bp.

**A APL B K562**

**
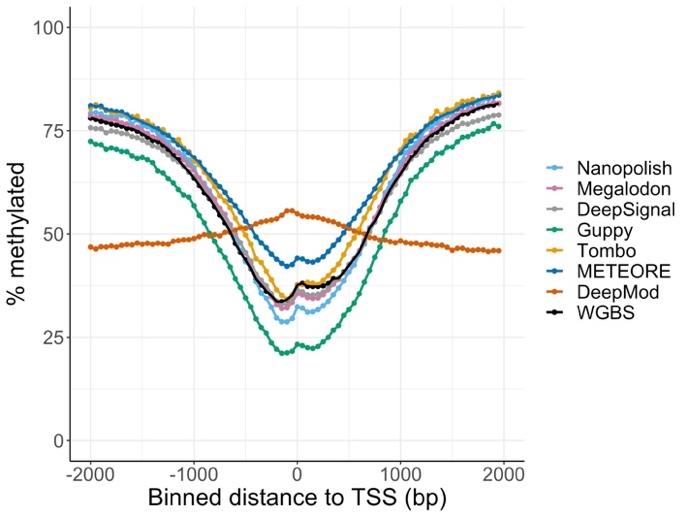

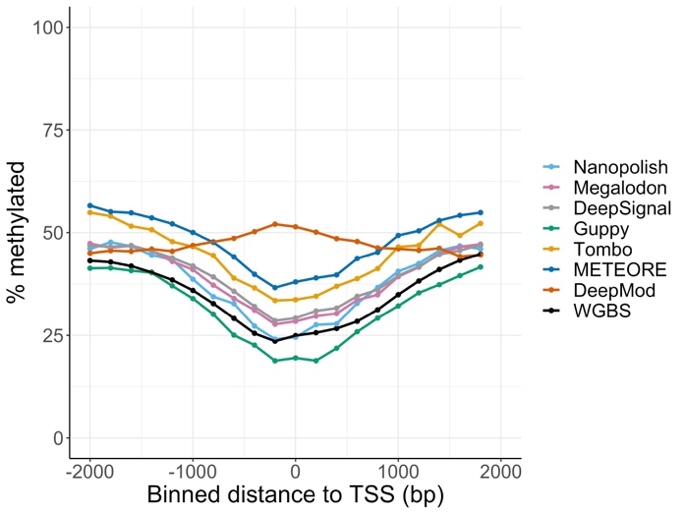
**

**C K562**

**
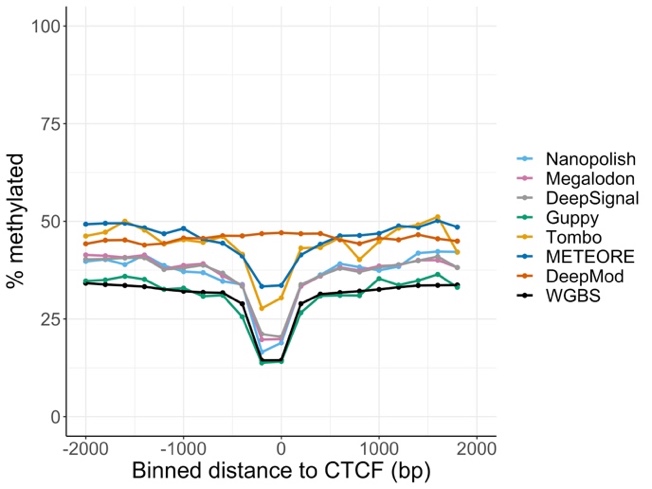
**

**Figure S7. Relationship between CpG methylation percentage and distance to annotated TSS and CTCF binding peaks.** (**A-B**) Relationship between CpG methylation percentage and distance to annotated TSS in (**A**) APL (bin size = 50 bp) and (**B**) K562 (bin size = 200 bp). (**C**) Relationship between CpG methylation percentage and distance to CTCF binding peaks in K562 with bin size = 200 bp.


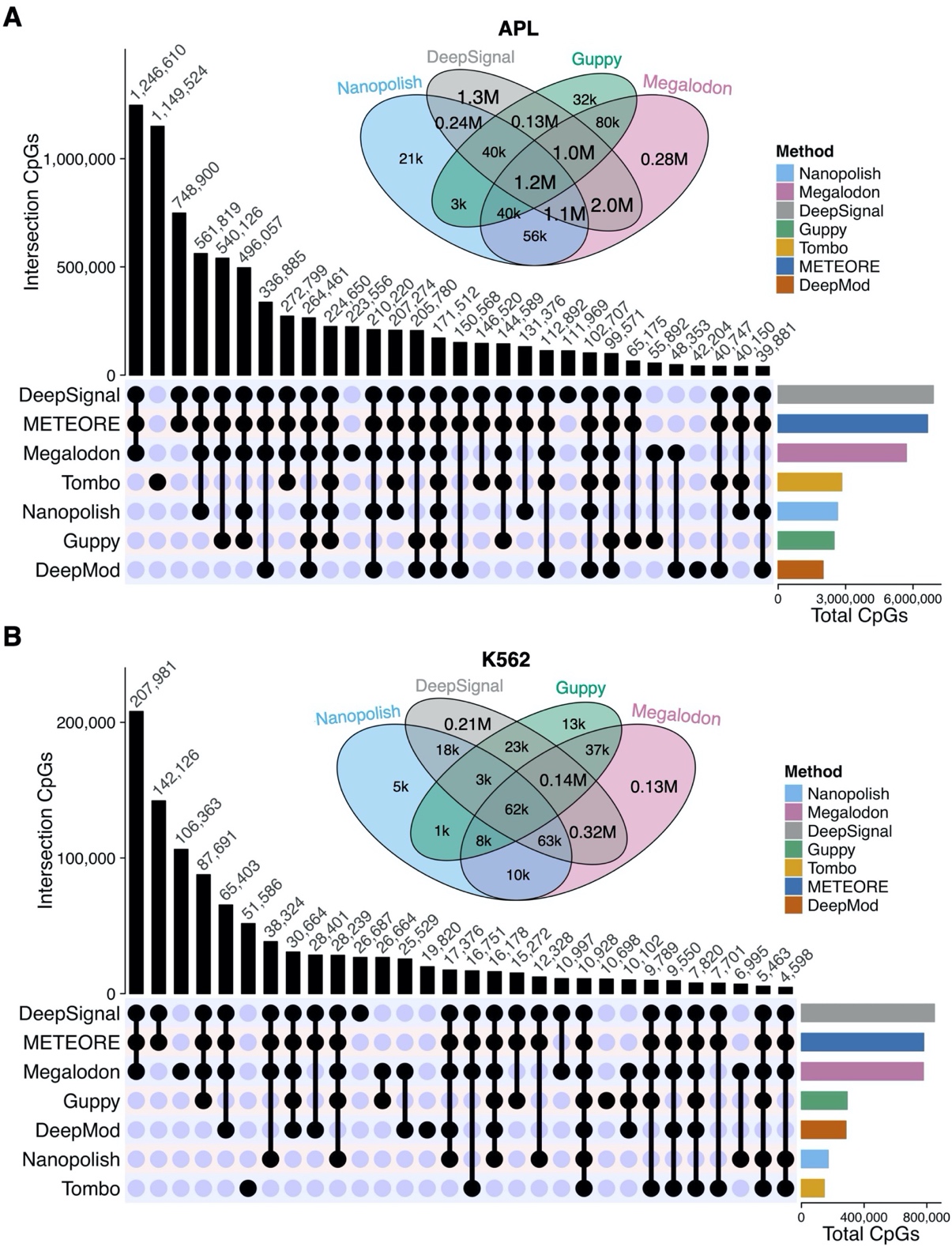


**Figure S8. The overlap of CpG sites predicted by methylation-calling tools using (A) APL and (B) K562 nanopore sequencing datasets.** UpSet diagram shown at bottom of each panel (**A** and **B**) is for the CpG sites of the top thirty sets of intersections detected by each methylation-calling tool, k is for thousand and M is for million. Venn diagram shown at the top of each panel (**A** and **B**) is for CpG sites detected by the four best-performing methylation-calling tools (Nanopolish, DeepSignal, Guppy, Megalodon). Bar plot shown at the lower right of each panel (**A** and **B**) is for the total CpGs detected by each tool. Only the CpG sites covered by **≥** 3 reads were considered for each tool.
