# Supplementary material for: DNA methylation-calling tools for Oxford Nanopore sequencing: a survey and human epigenome-wide evaluation
Source: Genome Biol. 2021 Oct 18;22:295. doi: 10.1186/s13059-021-02510-z (PMC8524990; doi:10.1186/s13059-021-02510-z)
Supplement: Supplementary file 3 — Additional file 3. Review history. [file 13059_2021_2510_MOESM3_ESM.docx]

Review History

**First round of review**

**Reviewer 1**

**Are you able to assess all statistics in the manuscript, including the appropriateness of statistical tests used?**

Yes, and I have assessed the statistics in my report.

**Comments to author:**

Liu et al. benchmarked five CpG methylation calling packages for Nanopore sequencing data. They systematically evaluated their performance in different aspects, which provides valuable information for researchers working on methylation profiling using ONT sequencing. However, in its current form, I have the following concerns which should be addressed.

Primary comments:

- Recent works have also benchmarked nanopore methylation calling software (e.g. 10.1186/s13059-021-02283-5, 10.1101/2020.10.14.340315), could the author stress their distinction in the current version compared to other studies?

- The author showed log-transformed read length in Fig2A, and claimed the read length of APL, K562, and HL-60 is comparable with the NA19240 dataset. This conclusion is arguable since the NA19240 tends to have a higher proportion of long reads (>10000 bp), this needs to be clarified, e.g. whether a different library preparation protocol was used.

- Fig3 showed all algorithms performed better on concordant non-singletons than singletons, can the author comment on that?

- Fig2 - Fig3, showed performance on singletons / non-singletons and genomic contexts. Is the performance on genomic context influenced by the distribution of singletons / non-singletons in those features?

- Figure 6 showed the percentage of covered CpG sites. All the tools use minimap2 for alignment, which means the covered CpG sites were the same. Could the author explain why the methylation tools will influence the covered CpG sites?

- Table 1 is missing

Minor comments:

- Recent works using bisulfite-free DNA methylation sequencing with Nanopore sequencing have shown that they provide high accuracy and do not require analytical tools (e.g. 10.1186/s13059-020-01969-6, 10.1101/gr.265306.120, 10.1093/nar/gkab397). It could be also added to the introduction about DNA methylation measurement.

- Figure2 A-B, D:

A: The y-axis should be labeled as read-length instead of log-transformed read length.

B: The y-axis should be labeled as the Base quality score instead of the Average base call quality score.

D: Two sub-figure is preferable since singletons / non-singletons and genomic contexts are two classification systems.

- Figure 5B-D, Figure S7 the data scale does not agree with the y-axis. If % methylation was used, the scale should be 0-100.

- There are a few figures got many fonts, eg. Figure3.

- Figure S1 typo (methylation)

**Reviewer 2**

**Are you able to assess all statistics in the manuscript, including the appropriateness of statistical tests used?**

Yes, and I have assessed the statistics in my report.

**Comments to author:**

Liu et al. present a comparison of 5 methylation-calling tools for fast5 sequence datasets produced by the Oxford Nanopore Technologies (ONT) third-generation sequencing platform. The authors propose a benchmarking workflow that uses 4 datasets, consisting of 3 cell lines and 1 clinical specimen. Each dataset was sequenced with ONT as the query data, and either WGBS or RRBS as ground truth data for assessing the methylation-calling tools. The ONT and WGBS/RRBS sequences were either publicly available, author-generated, or both depending on the dataset.

Evaluation of the tools consisted of three tests. Firstly, CpGs identified by WGBS or RRBS as having 0% or 100% methylation levels were compared to the per-read methylation calls of the 5 tools. The tools' set of predictions for each read covering a given CpG site could then be evaluated with confusion matrix measures against the associated WGBS/RRBS call at the CpG (precision, recall, accuracy, and F1 score). ROC AUC was further evaluated. The result of the confusion matrix measures at each CpG for each tool could then be compared in aggregate. Secondly, for remaining CpGs with > 0% and < 100% methylation called by WGBS/RRBS, Pearson's coefficients were calculated between each tool's predicted methylation status (per-site, not per-read). This also allowed for regression models to be created for predicted methylation at each CpG for pairs of the 5 tools. Thirdly, runtime and memory usage for each tool is compared under identical conditions. Overall, the authors found that one of the tools, Nanopolish, performs best in its agreement with WGBS/RRBS calls (both per-read and per-site), expected genomic context of methylation calls, and computational efficiency.

Synopsis:

This study provides a straightforward and descriptive comparison of nanopore based CpG methylation tools. Although the writing is rough in sections, and at times repetitive, the study provides a high-level review of nanopore sequencing technology advances along with step-by-step evaluation of the profiled tools that is likely to be of interest to those working in the area. The selection of a nanopolish as a 'preferred' tool in the conclusion of the results section is likely to be of specific interest, but this statement is confounded by the conclusion where is it suggested that nanopolish results should be 'integrated' with megalodon or deep signal output.

Comments

The statements regarding nanopolish vs. megalodon/deepsignal need to be clarified to provide the reader with a fulsome appreciation of the strengths and weakness of each. It is not clear how the results of the tools can be integrated or what this would accomplish. If the megalodon/deepsignal tools are essentially equivalent - but might be more suitable to a given compute architecture available to the researcher (high memory or not) - the authors should state this explicitly. If nanopolish provides a 'good enough' rapid assessment, but lacks the comprehensive analysis of megalodon/deepsignal - this should be stated.

The variety in the four datasets is a weakness of the comparisons as presented. Since most evaluations of the tools are within one dataset (apart from regression models), it is reassuring that relative ranking for each tool held between datasets. However, given the inherent variety in each dataset, many of the between-dataset comparisons have been modulated by multiple variables, both the unique attributes of the cell line/purified cells, and the different library preparation methods, sequencing types and depths performed on each. For example, the 'ground truth' correlation values shown in supplemental S5 demonstrate significant variability across and within the datasets presented and the rank order of correlations appears to be dataset dependent. This undermines the generalizability of the comparisons which is essential to assess the main conclusions of the manuscript.

It is surprising that one main advantage of nanopore sequencing is not discussed nor addressed in this study, the ability to distinguish hmC from mC and allele specific methylation. Might it be true, for example, that the increased correlations observed across nanopore callers, compared to BS-seq is driven in part by this? This might be addressed by examining the directionality of the methylation call differences between BS-seq and nanopore calling (ie Figure 5A)? Or even including a neuronal tissue type as part of the comparison.

Parts of the manuscript are repetitive, where the same analysis is discussed twice, albeit in slightly different contexts. i.e. the discussion of singletons and non-singleton analysis on pages 10 and 11. Figure 4A/B - Figure 5A - are not both comparisons performed against 'ground truth data" - just reporting different measures of comparison (F1 vs. correlation). These sections and others like it could be condensed.

The biological context of a given sequence does not impact methylation calling and such an analysis is obfuscated by the wide ranges of base compositions within classically defined 'biologically relevant genomic regions'. Rather than focusing on these measures, it might be useful to quantitatively extend the categories of singleton and non-singleton to include other sequence features, CG/AT densities, repetitive regions etc. This would provide a more quantitative comparison of the tools.

Minor comments:

The Venn diagram in Figure 6 is difficult to read. Authors should consider a Venn layout where non-overlapping, or partially overlapping portions, are represented as their own circles while preserving the color-coding to indicate relationships.

The dataset(s) analyzed in Fig 3 and 4 should be indicated in the legend and/or figure.

DeepMod should be included in the main figure set (exclusion noted in Page 11 line 41). Inclusion would be helpful to anyone using or considering using DeepMod that might read this paper.

The distinction between per-read and per-site evaluation could be made more explicit in the explanation of the first and second evaluation steps of the 5 tools for readers with little experience in ONT methylation calling. The latter is only implied in the explanation of the second evaluation method.

What is the meaning of "treated DNA were cleaned up" and "The final library was quantified by real time qPCR for an accurate concentration" at lines 44 and 49 on page 22?

On page 25, GRCh37 is incorrectly labelled as equivalent to hg37 rather than the more standard hg19 terminology

There is a typo in methylation in supplemental figure 1.

The points plotted in S4A/B are overlapping and thus impede interpretation. Smaller point sizes should be used.

In general, legends for the supplemental figures do not provide sufficient details to interpret the figures. 'Tool' should be moved to a different location in the figure - perhaps above.

**Authors Response**

**Point-by-point responses to the reviewers’ comments:**

Reviewer #1 (Reviewer comments to the author):

Liu et al. benchmarked five CpG methylation calling packages for Nanopore sequencing data. They systematically evaluated their performance in different aspects, which provides valuable information for researchers working on methylation profiling using ONT sequencing. However, in its current form, I have the following concerns which should be addressed.

*Response: We thank the reviewer for recognizing the potential interest of our work. We further thank the reviewer for the detailed and thoughtful critique. We have revised the manuscript to address these key points and believe that the work significantly benefited from this feedback.*

Primary comments:

1. Recent works have also benchmarked nanopore methylation calling software (e.g. 10.1186/s13059-021-02283-5, 10.1101/2020.10.14.340315), could the author stress their distinction in the current version compared to other studies?

*Response: We thank the reviewer for pointing out two other benchmarking papers on nanopore methylation-calling tools. Akbari et al., [1] focused primarily on methylation phasing using nanopore long reads, and compared only three DNA 5-methylcytosine (5mC)-calling tools (Nanopolish, Megalodon, DeepSignal) using one publicly available nanopore sequencing dataset: data generated from human lymphoblastoid cell line NA12878. Also, only a few genomic contexts (genic regions and CpG islands) and per-site performance (i.e., single-base resolution) were included in the evaluation of methylation calling.*

*Yuen et al. [2] trained on selected 100 CpGs, and performed benchmarking evaluation on a limited number of CpG sites: they selected 100 sites from E. coli and 1743 CpGs from NA12878 Cas9-targeted nanopore sequencing. They also developed a consensus approach named METEORE using machine learning models to combine predictions from two or more tools that shows improved accuracy over individual tools at limited number of CpG sites. Thus, we added the METEORE into our performance evaluation (Figure 3A, 4, 5A, Table S3) and introduced a published NA12878 dataset into our comparison since it is a well-established high-coverage nanopore sequencing dataset [3] and was also used as a benchmark dataset by Akbari et al., [1]. Therefore, thanks to the reviewers’ constructive feedback, our net improvement includes the following five aspects.*

*First, we presented a systematic benchmark of all seven current available methylation-calling tools for 5mC detection from nanopore sequencing data using human natural DNA at the whole-genome scale, not only in a limited number of CpG sites. A whole human-genome scale comparison is critical, as we noticed that one of the tools, i.e., DeepMod, while it performed well for the bacteria genome, performs poorly at the human whole-genome level, which was not previously reported. Also, we showed that the performance of METEORE across the human genome was worse than that of Nanopolish, Megalodon, DeepSignal and Guppy at the genome-wide scale across four datasets, which is distinctive from the prior study by Yuen et al. [2].*

*Second, to compliment current publicly available benchmark datasets generated from human cell lines, we generated (1) two nanopore sequencing datasets: one is from a primary leukemia specimen, and another is from a human leukemia cancer cell line, and (2) matched whole-genome bisulfite sequencing (WGBS) and oxBS-seq datasets. The new datasets enable the evaluation of these tools in a primary human specimen, not only in human cell lines, and thus will provide guidance on the application of nanopore sequencing in clinical research. In total, we used four human datasets for benchmarking, which is more than other published benchmark studies.*

*Third, we evaluated the prediction robustness not only at a per-site level, but also at a per-read level, and considered more diverse human genomic contexts, e.g., singletons, discordant and concordant non-singletons, genic and intergenic regions, various CG density regions, repetitive regions, and transcriptional factors CCCTC-binding factor (CTCF) binding regions.*

*Fourth, we demonstrated that the 5hmC levels contribute, at least in part, to the discrepancy between BS-seq and nanopore sequencing (see manuscript at line 293, page 12 for more details).*

*Fifth, we also compared the number of CpGs predicted by each tool and the computational resources consumed by each tool. This is essential for guiding the design of computational analyses on HPC and cloud computing for large-scale human nanopore sequencing data. Even the raw fast5 data from a single library, e.g., NA12878, with ~26x coverage, can use over 30 TB of storage space and over hundreds to thousands of CPU hours.*

*We have added discussion on the differences between the present study and the above two previous benchmarking studies (10.1186/s13059-021-02283-5, 10.1101/2020.10.14.340315) in our Introduction at line 112, page 5, and Discussion section at line 443, page 18.*

2. The author showed log-transformed read length in Fig2A, and claimed the read length of APL, K562, and HL-60 is comparable with the NA19240 dataset. This conclusion is arguable since the NA19240 tends to have a higher proportion of long reads (>10000 bp), this needs to be clarified, e.g. whether a different library preparation protocol was used.

*Response: We thank the reviewer for this question. Indeed, two different sequencing kits were used for library preparation. The NA19240 library was prepared using a Ligation Sequencing Kit (SQK-LSK108, ONT), while the libraries of the other four nanopore sequencing datasets were prepared using a Rapid Sequencing Kit (SQK-RAD004, ONT). The Ligation Sequencing Kit is optimized for throughput and long reads, and NA19240 has a higher proportion of long reads (> 10,000 bp) than other datasets. We apologize for the omission of this aspect, and have added the clarification in the Results section at line 164, page 7, and added the library preparation protocol for NA19240 in the Method section at line 612, page 24.*

3. Fig3 showed all algorithms performed better on concordant non-singletons than singletons, can the author comment on that?

*Response: We thank the reviewer for this question. First, DeepMod, METEORE, and Nanopolish models were trained using ‘concordant’ sequences – completely methylated sequences and completely unmethylated sequences. The unmethylated training dataset for DeepSignal is also from ‘concordant’ sequences – whole-genome amplified nanopore reads. Therefore, the design of the training datasets likely contributes to the higher performance in concordant non-singletons. Second, Nanopolish jointly predicted multiple CpG sites in one non-singleton region by combining their log-likelihood ratios (LLR) and reported only on methylation state. Similarly, Tombo/Nanoraw (referred to as Tombo) [5] borrows the strength across multiple statistical tests at adjacent nucleotides for detecting methylation. Thus, the design of the algorithm to detect methylation for neighboring CpG sites might also contribute to their higher performance on concordant non-singletons than on singletons. ONT-developed model-based tools, i.e., Guppy and Megalodon, lack publications or preprints to describe the details of their approaches. We appreciate the reviewer’s question and have added the statement below in the Results section at line 225, page 9 to enhance the clarity*

4. Fig2 - Fig3, showed performance on singletons / non-singletons and genomic contexts. Is the performance on genomic context influenced by the distribution of singletons / non-singletons in those features?

*Response: We thank the reviewer for this question. To investigate the problem, we assessed the percentage of singletons and non-singletons for each genomic context (Table S6). In addition, we tested the significance of the correlation coefficient between F1 score achieved by each tool and non-singleton percentage, and the relationship was statistically significant (p-value < 0.05, correlation coefficients range from 0.286 to 0.423) for Nanopolish, DeepSignal and Guppy (Table S7).*

*These observations suggest that the various 5mC prediction performances across different genomic contexts are significantly influenced by the distribution of singletons and non-singletons in at least three tools. We appreciate the reviewer’s question and have added the statement below in the Results section at line 259 on page 11.*

5. Figure 6 showed the percentage of covered CpG sites. All the tools use minimap2 for alignment, which means the covered CpG sites were the same. Could the author explain why the methylation tools will influence the covered CpG sites?

*Response: We thank the reviewer for this question. Although the covered CpG sites are same after the alignment, each tool has its own criteria to make confident methylation predictions, which influence their output CpG site numbers. For example, Nanopolish assigned a log-likelihood ratio (LLR) for 5mC prediction, where a positive LLR indicates support for methylation. Specifically, the CpG sites are methylated when LLR > 2.0, while the CpG sites are unmethylated when the LLR < -2.0. Megalodon predicts the log probability for a specific site and only counts the base (modified or canonical) when the log probability is >0.8 toward the final aggregated output at per-site level. DeepSignal generates two probability values for each site at read level, one for methylated (prob_1) and another for unmethylated (prob_0); and a site is predicted as methylated when prob_1 > prob_0, while it is called as unmethylated when prob_1 ≤ prob_0. More details and other methods description can be found at line 659-727, page 26-29. Thus, the number of predicted CpG sites differs among the tools. We appreciate the reviewer’s question and, to enhance the clarity, we added an explanation of why these tools predicted different number of CpG sites when using the same alignment tool (see the Results section, line 332, page 14).*

6. Table 1 is missing.

*Response: We thank the reviewer for pointing out this issue and apologize for the omission of Table 1. We have added the Table 1 to the manuscript, on page 34.*

Minor comments:

7. Recent works using bisulfite-free DNA methylation sequencing with Nanopore sequencing have shown that they provide high accuracy and do not require analytical tools (e.g. 10.1186/s13059-020-01969-6, 10.1101/gr.265306.120, 10.1093/nar/gkab397). It could be also added to the introduction about DNA methylation measurement.

*Response: We thank the reviewer for this suggestion. We have added the bisulfite-free DNA methylation sequencing method into text in the Introduction section that discusses DNA methylation measurement. Please see line 116, page 5 below.*

8. Figure2 A-B, D:

A: The y-axis should be labeled as read-length instead of log-transformed read length.

B: The y-axis should be labeled as the Base quality score instead of the Average base call quality score.

D: Two sub-figure is preferable since singletons / non-singletons and genomic contexts are two classification systems.

*Response: We thank the reviewer for these suggestions. We updated Figure 2 following the specific instruction.*

9. Figure 5B-D, Figure S7 the data scale does not agree with the y-axis. If % methylation was used, the scale should be 0-100.

There are a few figures got many fonts, eg. Figure3.

*Response: We thank the reviewer for these suggestions. We have updated the y-axis of Figure 5B-C and Figure S7 using a scale range from 0 to 100. In addition, we checked and replotted all figures using the same font, including Figure 3 and Figure 5A.*

10.Figure S1 typo (methylation).

*Response: We thank the reviewer for pointing out the typo. We have fixed the typo so that the text in Figure S1 (Figure 1B in resubmission) is now “methylation”.*

Reviewer #2:

Liu et al. present a comparison of 5 methylation calling tools for fast5 sequence datasets produced by the Oxford Nanopore Technologies (ONT) third-generation sequencing platform. The authors propose a benchmarking workflow that uses 4 datasets, consisting of 3 cell lines and 1 clinical specimen. Each dataset was sequenced with ONT as the query data, and either WGBS or RRBS as ground truth data for assessing the methylation calling tools. The ONT and WGBS/RRBS sequences were either publicly available, author-generated, or both depending on the dataset.

Evaluation of the tools consisted of three tests. Firstly, CpGs identified by WGBS or RRBS as having 0% or 100% methylation levels were compared to the per-read methylation calls of the 5 tools. The tools' set of predictions for each read covering a given CpG site could then be evaluated with confusion matrix measures against the associated WGBS/RRBS call at the CpG (precision, recall, accuracy, and F1 score). ROC AUC was further evaluated. The result of the confusion matrix measures at each CpG for each tool could then be compared in aggregate. Secondly, for remaining CpGs with > 0% and < 100% methylation called by WGBS/RRBS, Pearson's coefficients were calculated between each tool's predicted methylation status (per-site, not per-read). This also allowed for regression models to be created for predicted methylation at each CpG for pairs of the 5 tools. Thirdly, runtime and memory usage for each tool is compared under identical conditions. Overall, the authors found that one of the tools, Nanopolish, performs best in its agreement with WGBS/RRBS calls (both per-read and per-site), expected genomic context of methylation calls, and computational efficiency.

Synopsis:

This study provides a straightforward and descriptive comparison of nanopore based CpG methylation tools. Although the writing is rough in sections, and at times repetitive, the study provides a high-level review of nanopore sequencing technology advances along with step-by-step evaluation of the profiled tools that is likely to be of interest to those working in the area. The selection of a nanopolish as a 'preferred' tool in the conclusion of the results section is likely to be of specific interest, but this statement is confounded by the conclusion where is it suggested that nanopolish results should be 'integrated' with megalodon or deep signal output.

*Response: We are grateful to the reviewer for appreciating our manuscript. We also thank the reviewer for the detailed the thoughtful critique. We regret the repetitive writing and confusion in the conclusion. We have worked with Dr. Stephen Sampson, a scientific writer at JAX, to improve the writing and reduce the amount of repetition. We have also revised the manuscript to address these key points and believe that the work significantly benefited from this feedback.*

Comments

1. The statements regarding nanopolish vs. megalodon/deepsignal need to be clarified to provide the reader with a fulsome appreciation of the strengths and weakness of each. It is not clear how the results of the tools can be integrated or what this would accomplish. If the megalodon/deepsignal tools are essentially equivalent - but might be more suitable to a given compute architecture available to the researcher (high memory or not) - the authors should state this explicitly. If nanopolish provides a 'good enough' rapid assessment, but lacks the comprehensive analysis of megalodon/deepsignal - this should be stated.

*Response: We agree with the reviewer that we should provide an explicit statement on the strengths and weakness of each tool and the recommendation of each tool. To clarify the strengths and weakness of each tool, we generated a heatmap to summarize the performances of all tools across all major evaluation criteria and provide a systematic comparison (Figure 8):*

*For users with limited computational resources, we recommend Guppy and Nanopolish. Guppy requires minimum CPU hours and peak memory as one of the top four performers because the base-modification prediction is part of its base calling. Nanopolish is the best option considering per-read and per-site performance criteria, as well as low CPU hours and peak memory usage after base-calling.*

*For users with the access to large high-performance computing (HPC) resources or a larger budget for cloud computing resources, Megalodon is the best option, considering its performance in the more challenging areas including repetitive regions and discordant non-singletons, also as it predicts more CpG sites compared to Nanopolish and Guppy. See more details in our Conclusion section at line 515, page 21.*

2. The variety in the four datasets is a weakness of the comparisons as presented. Since most evaluations of the tools are within one dataset (apart from regression models), it is reassuring that relative ranking for each tool held between datasets. However, given the inherent variety in each dataset, many of the between-dataset comparisons have been modulated by multiple variables, both the unique attributes of the cell line/purified cells, and the different library preparation types and depths performed on each. For example, the 'ground truth' correlation values shown in supplemental S5 demonstrate significant variability across and within the datasets presented and the rank order of correlations appears to be dataset dependent. This undermines the generalizability of the comparisons which is essential to assess the main conclusions of the manuscript.

*Response: We thank the reviewer for this comment. We regret the confusion caused by how we present the results across datasets. We agree that the inclusion of the HL-60 (as shown in Figure S5C in original submission), i.e., the dataset with the lowest sequencing depth, greatly contribute to the heterogeneity of the benchmark datasets. Thus, we removed the HL-60 dataset from the current manuscript. We are grateful to the reviewer for appreciating that most of our evaluations are within individual datasets. Indeed, the ‘ground truth’ correlation value shown in Figure S5 of original submission demonstrate high variability within and across datasets. The variability among different tools within the same dataset is high, as the tools that perform poorly, e.g., DeepMod, generate output differ significantly from top performers. In the original submission, given that the across-dataset variability may potentially confuse the readers, we have removed Supplemental Figure 5 on correlation plot. Currently, we include the two large datasets with high coverage, i.e., NA12878 and NA19240, to cover two different kinds of library protocols that users may choose to use when they design their own analytic plan. We also include low-coverage datasets for users to guide their choice of analytic tools, and the matched oxBS-seq and WGBS datasets allow us to explore the ability of distinguishing 5hmC and 5mC by nanopore sequencing, as suggested by the reviewer in the next comment. Since both ONT sequencing library kits are widely available, for users seek to explore the performance of tools for each of the kit, we present the analytic results in Table S8. We are grateful to the feedback from the review. We believe the modifications we made following the reviewer’s comment significantly improve the clarity of the manuscript.*

3. It is surprising that one main advantage of nanopore sequencing is not discussed nor addressed in this study, the ability to distinguish hmC from mC and allele specific methylation. Might it be true, for example, that the increased correlations observed across nanopore callers, compared to BS-seq is driven in part by this? This might be addressed by examining the directionality of the methylation call differences between BS-seq and nanopore calling (ie Figure 5A)? Or even including a neuronal tissue type as part of the comparison.

*Response: We agree and thank the reviewer for the suggestions. We regret the omission of a thorough discussion of the ability to distinguish 5hmCs from 5mCs and allele-specific methylation by nanopore sequencing. Based on the reviewer’s feedback, we now added the discussion on the main advantages of nanopore sequencing and the new results from 5hmC data analysis.*

*5mC and 5hmC affect the electronic currents differently in the pore when DNA passes through: 5mC consistently increases the current relative to C, while 5hmC generally decreases the current relative to C, which reveals the potential feasibility to discriminate 5hmC, 5mC, and 5C by electric signal deviations [4, 5]. However, very few computational methods are currently available that can predict 5hmC from nanopore reads. SignalAlign [6] achieved a median accuracy of 76% for the performance in discriminating cytosine variants with a three-way (C, 5mC, or 5hmC) cytosine classification experiment using synthetic oligonucleotides, but the method is developed with nanopore chemical version R7.3 (the pore is out-of-date and no longer available) and its repository has not been updated for over four years. We also noticed that ONT sequencing recently provided a "research release" basecalling model trained in 5hmC and 5mC in all contexts (res_dna_rR941_min_modbases_5mC_5hmC_v001.cfg) in the Rerio repository [7], and Megalodon [8] may be able to utilize the 5hmC model to call 5hmCs and 5mCs simultaneously. However, the 5hmC model of Megalodon is quite preliminary and experimental, and to date no data are available on the model performance to evaluate 5hmC, 5mC and 5C at single-base resolution. For allele-specific methylation (ASM), nanopore sequencing is ideal to detect and phase ASM with its modification detection ability and long reads sequencing, For example, Akbari et al. [1] have developed a workflow and associated software to detect ASM in both human and B-lymphocyte cell lines (NA19240) or Colo829BL B-lymphoblast cell lines using nanopore sequencing data only. When more computational tools emerge for simultaneous prediction of multiple modifications (e.g., 5hmC and 5mC) from single read, these tools will likely expand the ability of allele-specific modifications detection by nanopore sequencing. We have added this part to the Discussion section; please check line 418, page 17.*

*Indeed, the correlation among the top four performers, i.e., Guppy, Nanopolish, Megalodon, and DeepSignal, is greater than the correlation between BS-seq and nanopore sequencing data. As suggested by the reviewer, we hypothesize that the ability to distinguish 5hmC from 5mC by nanopore sequencing, contribute to the discrepancy between BS-seq and nanopore sequencing. Thus, to test the hypothesis, we need the matched nanopore sequencing data, BS-seq, and 5hmC profiling from the same sample. And we agree that neuronal tissue will be ideal for 5hmC profiling. Although we could not find any publicly available datasets with matched nanopore sequencing data, BS-seq data, and 5hmC profiling by next-generation sequencing from neuronal tissue type, we generated matched oxBS-seq for APL. Therefore, we detected the 5hmC percentage for each CpG site by integrating matched APL oxBS-seq and WGBS data by the MLML (maximum likelihood methylation levels) algorithm [9]. We then compared the 5hmC percentage in CpGs exhibiting agreement (methylation level difference < 5%) and discrepancy (methylation level difference > 40%) between WGBS and nanopore sequencing for APL. The CpG sites exhibiting discrepancy showed a significantly higher 5hmC level than those exhibiting agreement (p-value < 0.0001, Wilcoxon rank sum test, Figure S6A-D). These observations suggest that the ability to distinguish 5hmC from 5mC by nanopore sequencing enables a more accurate 5mC detection than BS-seq. More details can be found at line 293 on page 12.*

4. Parts of the manuscript are repetitive, where the same analysis is discussed twice, albeit in slightly different contexts. i.e. the discussion of singletons and non-singleton analysis on pages 10 and 11. Figure 4A/B - Figure 5A - are not both comparisons performed against 'ground truth data" - just reporting different measures of comparison (F1 vs. correlation). These sections and others like it could be condensed.

*Response: We agree and thank the reviewer for the suggestions. We regret the repetitive text, which burdens reading. We have now condensed the text and the figures and clearly explain the differences of different sections. Importantly, we worked with the Dr. Stephen Sampson, a scientific writer at JAX, to remove the repetitive sentences and context as pointed out by the reviewer and throughout the manuscript.*

*First, for the repetitive text on pages 10-11 in the original submission, we combine and condensed two sections – “Predictions of 5mC at single-molecule, single-base resolution” (originally on page 9) and “Predictions of 5mC at single-molecule, single-base resolution across various biologically relevant genomic regions” (originally on page 10) into one section – “Per-read performance of 5mC prediction” (now at line 213 at page 9).*

*Second, F1 score vs. correlation related analysis both were using BS-seq as ground truth; F1 score assesses the per-read performance, while correlation assesses the per-site performance. We plotted the overall strategy in Figure 1C and describe the two types of performance, i.e., per-read and per-site performance, at line 185 on page 8. We apologize for the confusion due to the unclear writing in the Results section regarding Figure 5A on pages 10-11 in the original submission, which did not establish the logical flow clearly with respect to the overall strategy. We realized that the distinction between the title of original section – “Methylation calls by Nanopolish, Megalodon, and DeepSignal show high concordance with ground truth BS-seq” (originally on page 11) and the previous section titles are unclear. Thus, we revised the section title to “Per-site performance of 5mC prediction” at line 270, page 11.*

*Third, all the results of the different evaluation matrices including accuracy are listed in Table S3. Thus, we kept the figures on F1-scores and removed any figures on accuracy (Figure 4 and S4) to ameliorate the redundancy.*

*Together, these changes aim to condense multiple sections and emphasize the logic connections between benchmark strategy and results, so as to enhance the readability of the manuscript based on the reviewer’s thoughtful feedback.*

5. The biological context of a given sequence does not impact methylation calling and such an analysis is obfuscated by the wide ranges of base compositions within classically defined 'biologically relevant genomic regions'. Rather than focusing on these measures, it might be useful to quantitatively extend the categories of singleton and non-singleton to include other sequence features, CG/AT densities, repetitive regions etc. This would provide a more quantitative comparison of the tools.

*Response: We thank the reviewer for the suggestions. We regret the omission of performance evaluation in other sequence features. As advised, we have added the performance evaluation in regions of various CG density levels and repetitive regions; please find the updated results in Figure 4B-C, S4B-C below. Specifically, CG density was calculated by the percentage of G and C bases in 5-base windows, and therefore we compared different window sizes (20%, 40%, 60%, 80% and 100%); repetitive regions were categorized into short interspersed nuclear elements (SINE), long interspersed nuclear elements (LINE), long terminal repeats (LTR), DNA transposons, and “other” repetitive regions for comparison. We discuss the performance of tools in these regions at line 238, page 10, in the Results section.*

6. The Venn diagram in Figure 6 is difficult to read. Authors should consider a Venn layout where non-overlapping, or partially overlapping portions, are represented as their own circles while preserving the color-coding to indicate relationships.

*Response: We agree and thank the reviewer for the suggestion. We regret the lack of clarity in the Venn diagram in Figure 6. We replotted it to display non-overlapping or partially overlapping portions clearly on their own and use color-coding to indicate relationships as suggested. We also make sure that the numbers in each intersection are clear to read. Thus, we updated Figure 6, S8 accordingly. Please see the Venn Diagram from the updated Figure 6A.*

7. The dataset(s) analyzed in Fig 3 and 4 should be indicated in the legend and/or figure.

*Response: We thank the reviewer for the suggestion. We updated the title and legend of Figure 3 and 4 to indicate that the results are across four datasets: see line 858 - 868 on page 36.*

8. DeepMod should be included in the main figure set (exclusion noted in Page 11 line 41). Inclusion would be helpful to anyone using or considering using DeepMod that might read this paper.

*Response: We agree and thank the reviewer for the suggestion. We have included DeepMod into the main figures and supplemental figures (Figure 3-8, S2-8).*

9. The distinction between per-read and per-site evaluation could be made more explicit in the explanation of the first and second evaluation steps of the 5 tools for readers with little experience in ONT methylation calling. The latter is only implied in the explanation of the second evaluation method.

*Response: We thank the reviewer for this suggestion. We rewrote Step 3 of the overall strategy section to explicitly state that we evaluated (1) per-read and (2) per-site performance (please see line 185 - 195 on page 8).*

*We appreciate the feedback from the reviewers. We believe that the enhanced clarity in the overall strategy section will help readers who have little experience in ONT methylation detection to better understand the results of per-read and per-site performance evaluation in the next sections.*

10. What is the meaning of "treated DNA were cleaned up" and "The final library was quantified by real time qPCR for an accurate concentration" at lines 44 and 49 on page 22?

*Response: We thank the reviewer for this question. We regret the lack of clarity in this description. "Treated DNA were cleaned up" indicates that DNA was purified from the chemicals used in bisulfite treatment. "The final library was quantified by real time qPCR for an accurate concentration" is required because accurate quantitation is needed for loading the sequence for nanopore sequencing. We have added the explanation to the Methods section at line 573 on page 23.*

11. On page 25, GRCh37 is incorrectly labelled as equivalent to hg37 rather than the more standard hg19 terminology

*Response: We agree and thank the reviewer for the suggestion. We updated this to “GRCh37/hg19”. Please see text at line 647 on page 26.*

12. There is a typo in methylation in supplemental figure 1.

*Response: We thank the reviewer for pointing out the typo. We have fixed the typo and now use “methylation” and move this figure to Figure 1B.*

13. The points plotted in S4A/B are overlapping and thus impede interpretation. Smaller point sizes should be used.

*Response: We agree with the reviewer that the overlapping points impede interpretation. We have replotted the points using a smaller size. We thank the reviewers for the suggestion to improve the clarity of the plots. Please see updated Figure S3A-B.*

14. In general, legends for the supplemental figures do not provide sufficient details to interpret the figures. 'Tool' should be moved to a different location in the figure - perhaps above.

*Response: We thank the reviewer for these suggestions. We added detailed explanations in legends for all supplementary figures. We moved 'Tool' in all figures on the top location of the plot in Figure S3 and S4. Please see supplementary figures and figure legends.*

*Reference*

*1. Akbari V, Garant J-M, O' N, Kieran, Pandoh P, Moore R, Marra MA, Hirst M, Jones SJM: Megabase-scale methylation phasing using nanopore long reads and NanoMethPhase. Genome Biology 2021, 22:1-21.*

*2. Yuen ZW-S, Srivastava A, Daniel R, McNevin D, Jack C, Eyras E: Systematic benchmarking of tools for CpG methylation detection from nanopore sequencing. Nature Communications 2021, 12:1-12.*

*3. Jain M, Koren S, Miga KH, Quick J, Rand AC, Sasani TA, Tyson JR, Beggs AD, Dilthey AT, Fiddes IT, et al: Nanopore sequencing and assembly of a human genome with ultra-long reads - Nature Biotechnology. Nature Biotechnology 2018, 36:338-345.*

*4. Laszlo AH, Derrington IM, Brinkerhoff H, Langford KW, Nova IC, Samson JM, Bartlett JJ, Pavlenok M, Gundlach JH: Detection and mapping of 5-methylcytosine and 5-hydroxymethylcytosine with nanopore MspA. Proceedings of the National Academy of Sciences 2013, 110:18904-18909.*

*5. Schreiber J, Wescoe ZL, Abu-Shumays R, Vivian JT, Baatar B, Karplus K, Akeson M: Error rates for nanopore discrimination among cytosine, methylcytosine, and hydroxymethylcytosine along individual DNA strands. Proceedings of the National Academy of Sciences 2013, 110:18910-18915.*

*6. Rand AC, Jain M, Eizenga JM, Musselman-Brown A, Olsen HE, Akeson M, Paten B: Mapping DNA methylation with high-throughput nanopore sequencing. Nature Methods 2017, 14:411-413.*

*7. Oxford Nanopore Technologies: rerio. 2021.*

*8. Oxford Nanopore Technologies: Megalodon. 2020.*

*9. Qu J, Zhou M, Song Q, Hong EE, Smith AD: MLML: consistent simultaneous estimates of DNA methylation and hydroxymethylation. Bioinformatics 2013, 29:2645-2646.*

**Second round of review**

**Reviewer 1**

In the added table 1, there are some footnotes missing (i.e. for g-i), other than that, I'm happy with the revision.

**Reviewer 2**

This reviewer thanks the authors for their considered and detailed response to initial review. This reviewer is satisfied with the authors responses and revisions to the initial critiques. As the authors note, in the intervening period between reviews a similar benchmarking study was published by Yuen et al., along with a consensus caller “METEORE” which the authors now include in their revised version. While these studies appear similar, the present study provides a more comprehensive analysis of methylation in a variety of sequence contexts, and indeed appears to have revealed a potential weakness in the METEORE tool that will require additional follow-up by the research community. This reviewer commends the authors for providing positive guidance for tool application in their concluding statements also summarized in figure 8.
